# Impact of Heat Stress on Cellular and Transcriptional Adaptation of Mammary Epithelial Cells in Riverine Buffalo (*Bubalus Bubalis*)

**DOI:** 10.1371/journal.pone.0157237

**Published:** 2016-09-28

**Authors:** Neha Kapila, Ankita Sharma, Amit Kishore, Monika Sodhi, Pawan K. Tripathi, Ashok K. Mohanty, Manishi Mukesh

**Affiliations:** 1 ICAR-National Bureau of Animal Genetic Resources, Karnal-132001, Haryana, India; 2 Singhania University, Jhunjhunu, Rajasthan, India; 3 ICAR-National Dairy Research Institute, Karnal-132001, Haryana, India; University of Colorado, School of Medicine, UNITED STATES

## Abstract

The present study aims to identify the heat responsive genes and biological pathways in heat stressed buffalo mammary epithelial cells (MECs). The primary mammary epithelial cells of riverine buffalo were exposed to thermal stress at 42°C for one hour. The cells were subsequently allowed to recover at 37°C and harvested at different time intervals (30 min to 48 h) along with control samples (un-stressed). In order to assess the impact of heat stress in buffalo MECs, several *in-vitro* cellular parameters (lactate dehydrogenase activity, cell proliferation assay, cellular viability, cell death and apoptosis) and transcriptional studies were conducted. The heat stress resulted in overall decrease in cell viability and cell proliferation of MECs while induction of cellular apoptosis and necrosis. The transcriptomic profile of heat stressed MECs was generated using Agilent 44 K bovine oligonucleotide array and at cutoff criteria of ≥3-or ≤3 fold change, a total of 153 genes were observed to be upregulated while 8 genes were down regulated across all time points post heat stress. The genes that were specifically up-regulated or down-regulated were identified as heat responsive genes. The upregulated genes in heat stressed MECs belonged to heat shock family *viz*., HSPA6, HSPB8, DNAJB2, HSPA1A. Along with HSPs, genes like BOLA, MRPL55, PFKFB3, PSMC2, ENDODD1, ARID5A, and SENP3 were also upregulated. Microarray data revealed that the heat responsive genes belonged to different functional classes *viz*., chaperons; immune responsive; cell proliferation and metabolism related. Gene ontology analysis revealed enrichment of several biological processes like; cellular process, metabolic process, response to stimulus, biological regulation, immune system processes and signaling. The transcriptome analysis data was further validated by RT-qPCR studies. Several *HSP* (*HSP40*, *HSP60*, *HSP70*, *HSP90*, and *HSPB1*), apoptotic (*Bax* and *Bcl2*), immune (*IL6*, *TNFα and NF-kβ*) and oxidative stress (*GPX1* and *DUSP1*) related genes showed differential expression profile at different time points post heat stress. The transcriptional data strongly indicated the induction of survival/apoptotic mechanism in heat stressed buffalo MECs. The overrepresented pathways across all time points were; electron transport chain, cytochrome P450, apoptosis, MAPK, FAS and stress induction of HSP regulation, delta Notch signaling, apoptosis modulation by HSP70, EGFR1 signaling, cytokines and inflammatory response, oxidative stress, TNF-alpha and NF- kB signaling pathway. The study thus identified several genes from different functional classes and biological pathways that could be termed as heat responsive in buffalo MEC. The responsiveness of buffalo MECs to heat stress in the present study clearly suggested its suitability as a model to understand the modulation of buffalo mammary gland expression signature in response to environmental heat load.

## Introduction

Heat stress is a significant issue for many livestock enterprises, particularly for dairy. The negative impact of heat stress on dairy animals have been well documented [1–6] that includes reduced feed intake, milk production, milk quality, fertility and increased respiratory, heart rates, panting activity, peripheral blood flow, sweating etc. These impacts can result in significant loss of income and increase in management costs. Estimated average losses resulting from heat stress for the US dairy industry were about US$900 million per annum [7]. Such apprehensions have increased even more in recent years with the increased global warming and strive of dairies to maximize milk production.

In India, dairy industry is mainly dependent on cattle and buffaloes with buffaloes contributing more than 50% of milk production in addition to draught power and meat. Depending upon the habitat and chromosome number, the domesticated water buffaloes has been classified into two main categories, namely riverine and swamp. The riverine buffaloes (2n = 50) primarily developed for milk, meat and draught purposes, are mainly found in India and countries to the west of India like Pakistan, Iran, Iraq, Egypt, Bulgaria, Italy etc. The swamp buffaloes (2n = 48) on the other hand have been primarily developed for draught purposes and are mainly found in South East Asia and China. The riverine buffalo is established as an economically important species not only in India but in many Asian and Mediterranean countries and hence its genetic improvement ranks high amongst agricultural research needs of these countries.

Of the total world buffalo population, 97.1% buffaloes are distributed predominantly in Asian countries, while only 2.9% are found in rest of the world. The largest buffalo populated countries are India, Pakistan and China, of which India and Pakistan account for almost 65% of the total world buffalo population. India is bestowed with immense richness of buffalo diversity with over 96.9 million buffaloes constituting more than half (56.8%) of the total world (170.4 million) and 58.9% of Asian (165.4 million) buffalo population, respectively [8].

The role of buffaloes as a major milk producing species is well recognized in the Indian sub-continent, especially in India and Pakistan where 92% of the world buffalo’s milk is produced. With higher total solids (protein, fat, minerals) of 18–23% as compared to 13–16% in cow milk, buffalo milk confers advantage in the preparation of cheese, curd and other dairy products [8].

Although buffaloes are well suited to hot and humid climates and muddy terrain, but they exhibit signs of great distress when exposed to direct solar radiation or while working in the sun during hot weather. It is reported that milk yield, growth rate and fertility of buffaloes get reduced during periods of high ambient temperature [9]. This is due to the fact that buffaloes absorb a great deal of solar radiations because of their dark skin and sparse coat or hair. In addition to that they possess less efficient evaporative cooling system due to their rather poor sweating ability as buffalo skin has one-sixth of the density of sweat glands than that of cattle skin. Under heat stress, buffalo’s body temperature, pulse rate, respiration rate and general discomfort increase quickly [9]. It further evokes a series of drastic changes in biological functions including decreased intake efficiency and utilization of feed; disturbances in metabolism of water, protein; and changes in energy and mineral balances, enzymatic reactions, hormonal secretions and blood metabolites. All this result in impairment of growth, reproductive as well as productive performance and hence overall well-being [10].

Even though buffaloes play a major role in sustaining the economy of India’s agriculture, still the full genetic potential of this species has not yet been well exploited, primarily due to lack of basic data on buffalo genome. The progress in the development of cattle, pig, sheep and chicken genome database continues worldwide, but similar efforts for buffalo are relatively of lesser magnitude. Previously, efforts have been made understand response of buffalo to heat stress on the basis of anatomical differences and physiological parameters; however, genetic components, gene regulatory pathways, alterations in gene expression and molecular mechanisms underlying changes in heat stress response are not well established in this species. The present scenario calls for mining of genomic data on this important genetic resource to understand about the genes, pathways and molecular mechanism of heat stress response. Microarray technology proven to be a powerful tool for the simultaneous expression analysis of thousands of genes in a tissue could be the most appropriate tool for this purpose [11].

Heat stress is an important environmental stimulus that can affect the expression of mRNA in different animal tissues or cells. Mammary gland, an important economical organ of dairy animals has always drawn attention of the scientists for over a century because of special function for milk synthesis and secretion. To understand the physiological function of mammary gland, mammary tissue cells or explants have been widely-used as *in vitro* models. However, when using tissue explants, it is inherently difficult to distinguish between primary mitogens and secondary regulators of mammary gland function and development. To circumvent most of these difficulties, emphasis has been placed on cell culture methodologies to study growth regulation, hormonal responsiveness, or biochemical properties of mammary epithelial cells [12]. Mammary epithelial cells (MECs) are responsible for converting most precursors into milk constituents and transporting them to the mammary lumen, the first line that gets affected by heat stress. As MEC are the predominant cell types in lactating mammary gland, changes in their genes expression could provide an insight of the mammary gland mechanism. Hence buffalo MECs could be an interesting *in-vitro* model to delineate the genes whose expression is significantly modulated due to heat stress challenge. To the best of our knowledge, no systematic initiative has been attempted so far to highlight the molecular mechanism or identify gene networks and molecular pathways associated with heat stress response in buffaloes using MEC. Transcriptomic adaptations of buffalo mammary epithelial cells during heat stress can be easily and efficiently identified utilizing bovine arrays. Considering the above issues, the present study was planned to generate global expression profile of buffalo MECs during normal and heat stressed state and identify molecular pathways significantly regulated in heat stressed MECs

## Material and Methods

### Buffalo MECs primary culture and heat treatment

The buffalo mammary gland tissues of approximately 5gm were obtained from a healthy adult buffalo from Gazipur abattoir 28.734190N and 77.272830E, New Delhi, India. The primary MECs were cultured using DMEM/F12, supplements and growth conditions as described earlier [13]. After several passages, 80% confluent buffalo MECs on 10th passage were distributed in collagen treated 12-wellplates (Corning, USA) in two sets with one plate assigned as control (kept at 37°C all the time) and other plate as treated (exposed to 42°C). Initially, cells were incubated at 37°C with 5% CO_2_ to stabilize the culture. Subsequently, the plate marked as treated was exposed to 42°C for one hour to simulate heat stress (HS) condition. After 1h, the cells were allowed to recover at 37°C, 5% CO_2_and harvested by trypsinization at different time points (30m, 2h, 4h, 8h, 12h, and 24h). The samples from control (CTR) plates were also trypsinized and harvested at the same time points corresponding to the treated plates. Followed by exposure to heat stress, cell viability and growth characteristics of buffalo MECs in normal and heat treated samples were determined using commonly used trypan blue dye exclusion method.

### Estimation of cellular proliferation towards heat stress to MECs

The induction and inhibition of proliferation of buffalo MECs under normal and heat stress condition in *in vitro* model was evaluated using MTT assay kit (Cayman, Ann Arbor). Cells were seeded in triplicate with a density of 5x10^3^ cells/well in 100 μl of culture medium in 96 well plates (Corning, USA) and cultured for 24–48 h at 37°C, 5% CO_2_. Cells in control plates were maintained at 37°C, 5% CO_2_ throughout the time-course, while those in treatment plates were exposed at 42°C, 5% CO_2_ for 1 h and then shifted to 37°C, 5% CO_2_. The post heat treated cells were harvested at different time points (0h, 30 m, 2h, 4h, 6h, 8h, 12h, 24h and 48h) and cell proliferation assay was performed following manufacturer’s instructions.

### Cell apoptosis detection by flow cytometry

The effect of heat stress on cell apoptosis of buffalo MECs was determined using ANNEXIN V-FITC / 7-AAD Kit (Beckman Coulter, France). Buffalo MECs were cultured, heat stressed and harvested at different time points as mentioned previously. The cell apoptosis assay was conducted as per manufacturer’s instructions. For Annexin V-FITC, the maximum fluorescence absorption and emission were recorded at wavelength of 490nm and 525nm, respectively, whereas, for 7-AAD viability dye, the maximum fluorescence absorption and emission for DNA/7-AAD complex were recorded at 543 nm and 655 nm, respectively.

### Real-time quantitative PCR (qPCR)

Total RNA extraction from MECs harvested at CTR, 30m, 2h, 4h, 8h, 12h, 24h after heat stress and cDNA synthesis was performed as described in our previous studies [13]. Primer details for all the heat shock protein and apoptotic gene family are provided as supplementary information (S1 Table). The accuracy of primer pairs was also ensured by the presence of a unique peak during the dissociation step at the end of qPCR cycle. qPCR was performed using Light Cycler 480 instrument (Roche, Germany) as described in our previous reports [13]. The data was acquired using the ‘second derivative maximum’ method as computed by the Light Cycler Software 3.5 (Roche Diagnostics) and subjected for subsequent analysis.

#### Data Normalization

In our previous study, *RPL4*, *EEF1A1*and *RPS23* were observed to be most stably expressed genes in heat stressed buffalo MEC [13] and identified as appropriate panel of reference genes for normalization of qPCR data utilizing geNorm, NormFinder and Best keeper softwares [14–16] The geometric averages of these genes were f utilized for normalization of qPCR data generated in the present study. To measure the relative changes in gene expression data, 2^**-ΔΔCT**^ method was used [17].

### Generation of microarray based gene expression data

As a first step towards One-Color Microarray-Based Gene Expression Analysis using Low Input Quick Amp Labelling kit (Agilent Technologies, Santa Clara, CA), the RNA samples (CTR, 30 m, 2h, 4h, 8h, 1h, 24 h) were labelled with T7 promoter primer (65°C for 10 min followed by incubation in ice for 5 min). cDNA was constructed from labelled RNA samples after adding cDNA master mix (5X First Strand Buffer, 0.1 M DTT, 10 mM dNTP mix and Affinity Script RNase Block Mix) followed by incubation at 40°C for 2 h, 70°C for 15 min and final incubation on ice for 5 min. The cRNA synthesis and amplification was performed by adding transcription master mix (5X Transcription Buffer, 0.1 M DTT, NTP mix, T7 RNA Polymerase Blend and Cyanine 3-CTP) followed by incubation at 40°C for 2 h. The amplified labelled cRNA was purified (RNeasy mini column kit, Qiagen, Germany), quantified {μgcRNA yield = (Concentration of cRNA) x 30 μL (elution volume)/ 1000} and checked for specific activity {pmol Cy3 per μg cRNA = (Concentration of Cy3/ Concentration of cRNA) x 1000}. All the samples exhibited yield and specific activity values higher than the minimum value of 1.65 and 6, respectively using four pack microarray format. For hybridization, 1.65 μg of linearly amplified and cyanine 3-labeledcRNA were fragmented using fragmentation mix (10X Blocking Agent and 25X Fragmentation Buffer) and incubated at 60°C for exactly 30 min. The fragmented RNA samples were immediately transferred on ice for one minute and 55μl of 2x GEx Hybridization Buffer HI-RPM was added to stop the fragmentation reaction. The fragmented samples (110μl volume) were loaded to the array placed in slide chamber. The assembled slide chamber placed in rotisserie was put in a hybridization oven set to rotate at 10 rpm and 65°C. The hybridization was allowed for 17 hours. Followed by hybridization, microarray slide was disassembled in GE wash buffer 1 (pre warm overnight at 37°C) containing Triton X-102 (0.005%),washed with fresh GE wash buffer 1 for 1 min followed by second wash with GE Wash Buffer 2 for 5 min at room temperature. The slide was dried and scanned immediately.

#### Scanning, data acquisition and data analysis

Immediately after washing, the slides were scanned on GenePix-4000B (Molecular Device) microarray scanner using one colour scan setting with 5μm resolution at wavelength of 532 nm (Cy3). After scanning, the images were collected as 16 bit images and finally stored as tif image files. These files were further subjected to feature extraction using Agilent Feature Extractor Software Version 9.5 (Agilent Technologies) software. GeneSpring GX 12.6 (Agilent technologies) was used to perform the analysis of raw data obtained from feature extraction software. The data was normalized to the 75^th^ percentile. The microarray analysis workflow employed in the present study consisted of following steps *viz*., loading of raw data into GeneSpring software, normalization of microarray data, quality check of microarray data, filtration of probe sets, identification of differentially expressed genes based on fold change criteria, classification/clustering of genes, gene ontology enrichment analysis and pathway analysis.

## Results and Discussion

### Establishment of primary culture of buffalo MECs

**Fig 1:**
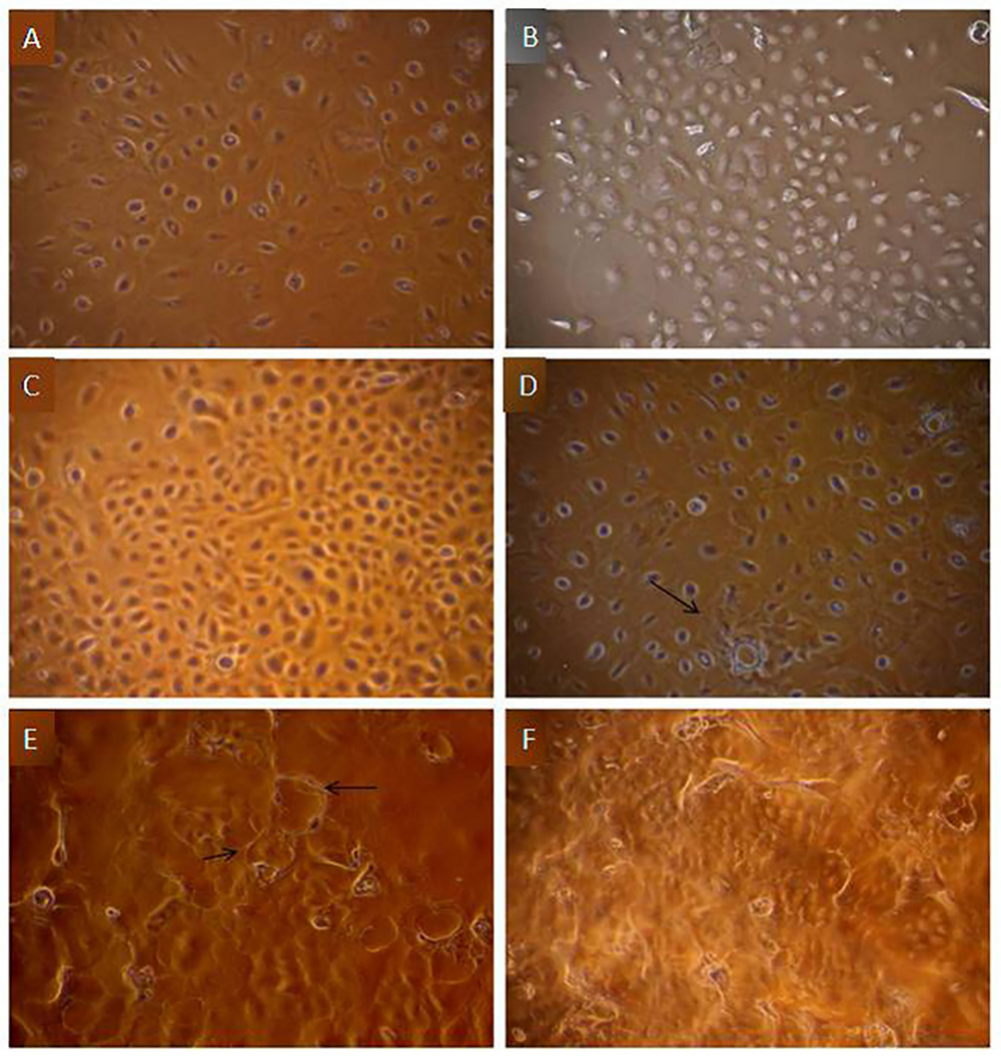
Phase-contrast images of buffalo MECs culture. A) Mixed population of epithelial and fibroblast cells, B) Formation of islands by purified MECs, at low density seeding, C) Cobble stone morphology shown by confluent MECs, D) Post confluent stage of MECs forming dome structures (arrow), E) MECs from passage 5 forming papillate structures, F) Floating dead cells due to occurrence of contact inhibition during post confluency stage.

The primary culture of buffalo mammary epithelial cells was achieved following enzymatic digestion of buffalo mammary tissue. In the initial stage heterogeneous population of epithelial and fibroblast-like cells was observed (Fig 1A) &that was further purified using selective trypsinization procedure. Being more sensitive to trypsin treatment as compared to MECs, fibroblasts cells were removed and homogeneous population of mammary epithelial cells was obtained. On microscopic evaluation the cells showed normal characteristics of MECs (Fig 1B–1E). Upon reaching the confluence stage (5–6 days after seeding), the cells formed a monolayer & aggregated to form typical cobble stone morphology of MECs (Fig 1C). During the post confluence period, a number of floating dead cells were observed indicating the occurrence of contact inhibition (Fig 1F) The MECs were passaged up to 10 times during continuous sub culturing. Following the similar methodology, primary MEC culture has been established in different livestock species [18–22] and in bovines, established MEC line has also been used to study the effect of heat stress *in vitro* [12, 23–26].

#### Immunofluorescent staining for assessing purity of cultured MECs

**Fig 2:**
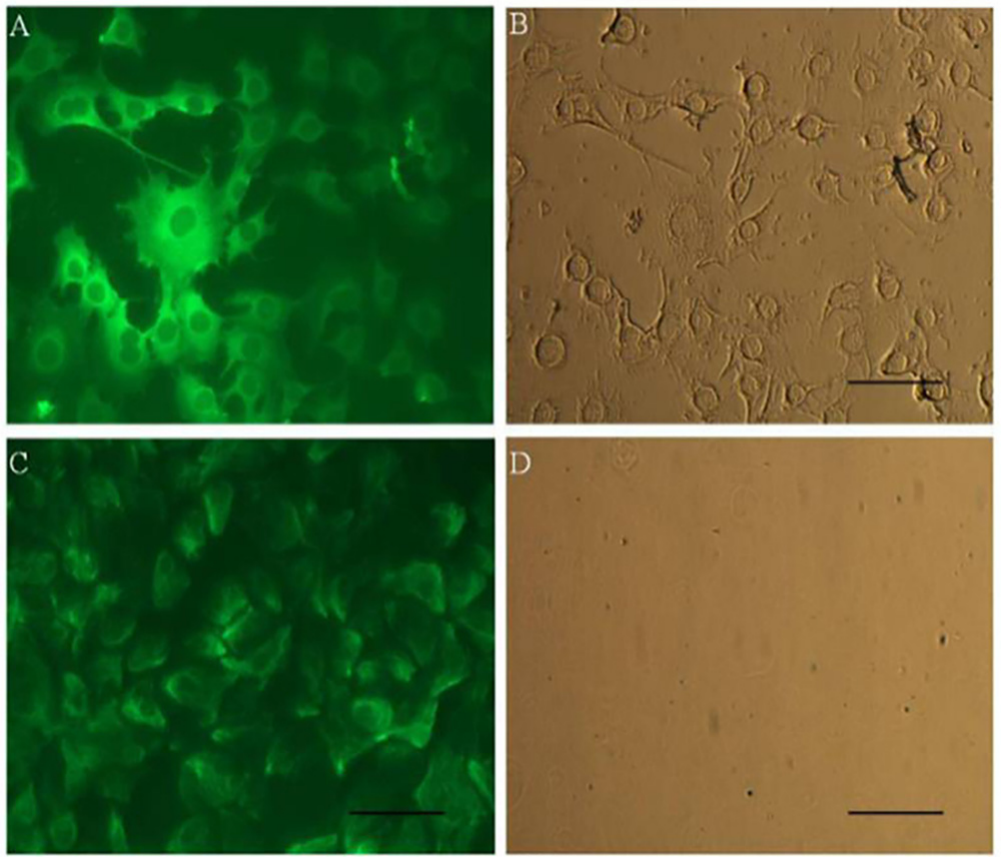
Immunocytostaining for expression of cytoskeletal markers in buffalo MECs A) Fluorescent image of Cytokeratin 18 showing intermediate filaments running in bundles with interconnections between cells, B) Light image of Cytokeratin 18, C) Fluorescent image of buffalo MECs stained for Vimentin showing filament degradation, D) Light image of Vimentin.

Purity of mammary epithelial cells was ensured by evaluating the expression of cytokeratin 18 and vimentin proteins using immune fluorescence staining. The anti-cytokeratin 18 antibody detects the expression of cytokeratins, known to be specific to epithelial cells whereas anti-vimentin antibody detects vimentin specific to fibroblast cells. The staining with anti- cytokeratin 18 antibody revealed strong signals indicating that high percentage of cells are of epithelial lineage (Fig 2A & 2B). Although, few cells were stained with anti-vimentin, but the signals were week (Fig 2C & 2D) with staining restricted mainly to the peripheral portion of the cytoplasm. It might be attributed to the presence of networks of intermediate filaments, important for cell to cell communication & polarity [20]. The immuno staining pattern with homogeneity and strong signal for cytokeratin 18, provided the evidence that the buffalo primary culture established in the present study mainly consisted of MECs with no or very little contamination of fibroblast cells. Our findings are in accordance with other reports across species, wherein it is reported that expression of cytoskeleton is specific for epithelial cells [19–20, 22, 27–32].

### Effect of heat stress on cell viability, proliferation, cytotoxicity and cellular apoptosis

**Fig 3:**
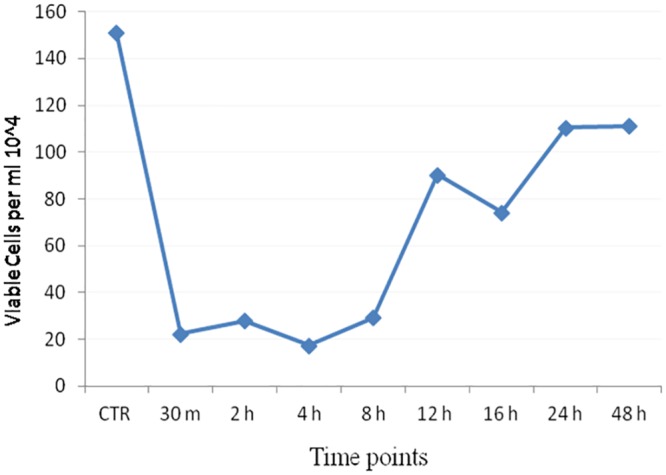
Cell viability pattern in heat stressed buffalo MECs using trypan blue dye exclusion method. CTR-unstressed (control) MECs.

In order to assess the effect of heat stress on MECs, cell viability & growth parameters were recorded using trypan blue dye, MTT assay & flow cytometric analysis. The cell viability estimation by trypan blue exclusion method showed decreased cell number and viability in heat stressed cells across different time points post heat stress (30 m, 2 h, 4 h, 6 h, 8 h, 12 h, 24 h & 48 h) whereas the unstressed (control) cells maintained at 37°C did not show reduction in cell viability (Fig 3). A decrease in cell viability was recorded immediately (30 m) after heat treatment and it remained significantly low up to 8 h of recovery phase. Thereafter, the cell viability increased during later stages of recovery (>12 h). The low percentage of viable cells observed immediately after the heat stress might be attributed to the adverse effect of heat stress on cell membrane structure or/and to cell necrosis or impairment of cell growth, while the recovery in cell viability during the later stages, could be attributed to the restoration of cell survival mechanism by mammary cells. The non-linear response of the cell viability to heat stress observed in the present study is in accordance with other studies where similar response of the cell viability to heat stress in different *in vitro* cell culture models was examined [23, 33–37].

**Fig 4:**
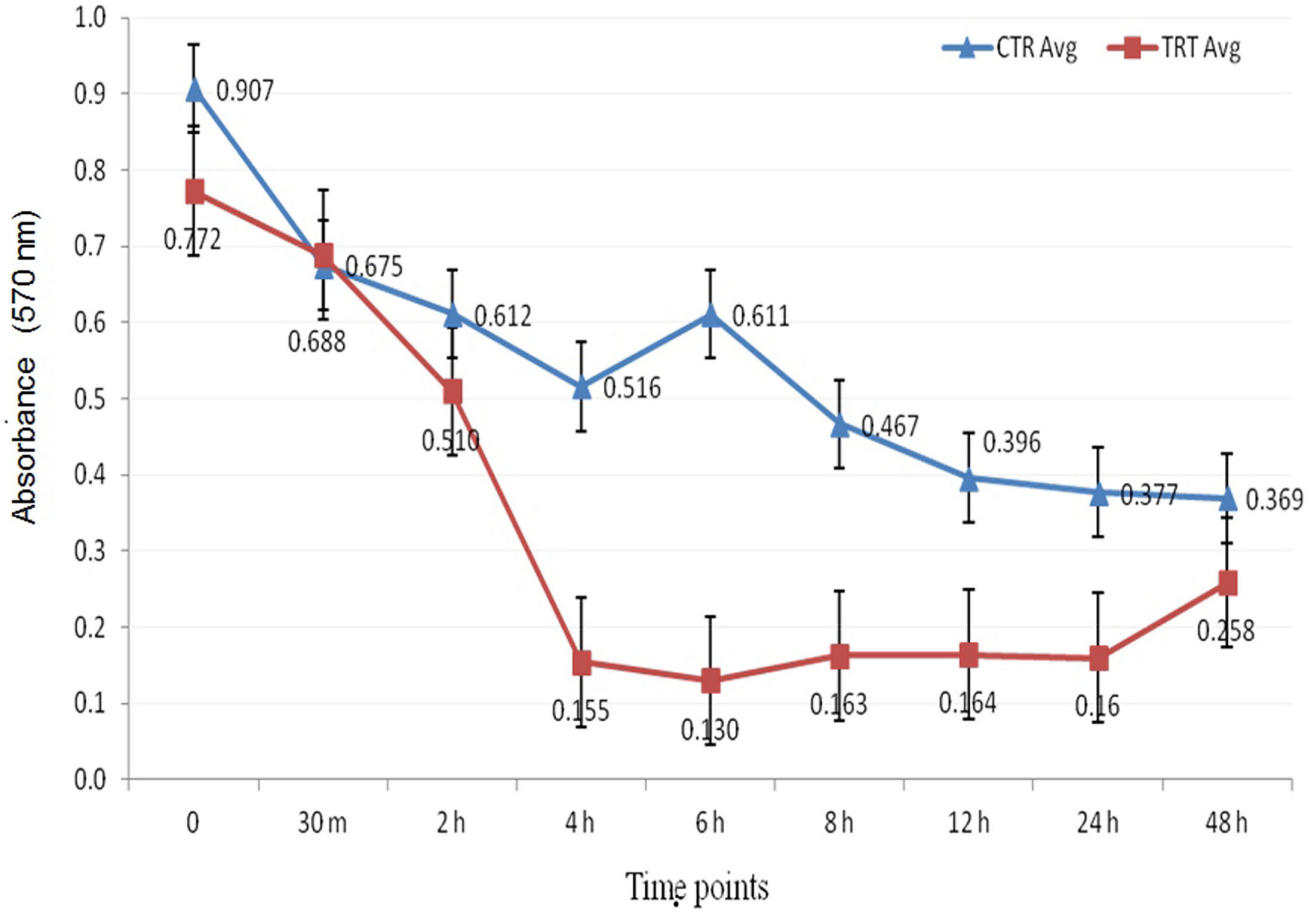
Evaluation of cellular proliferation in unstressed (control) and heat stressed buffalo MECs using MTT assay. CTR-unstressed (control); TRT- heat stress treated MECs.

Further, the effect of heat stress on cell viability & proliferation of MECs was also determined using MTT based assay. The heat stressed MECs(exposed to 42°C for 1 hour) had significantly lower cell viability than unstressed (control) cells. There was significant reduction in cell proliferation immediately after the heat stress & remained low throughout the time course (Fig 4). The lower formazan crystal formation in damaged cells indicated loss of cell proliferation efficiency in post heat stress samples as compared to unstressed cells. Hence decrease in cell proliferation efficiency in heat stressed buffalo MEC might be due to loss of plasma membrane potential. Similar observation has been reported in bovine MECs where thermal stress inhibits the proliferation rate [12, 24,36,38].

**Fig 5:**
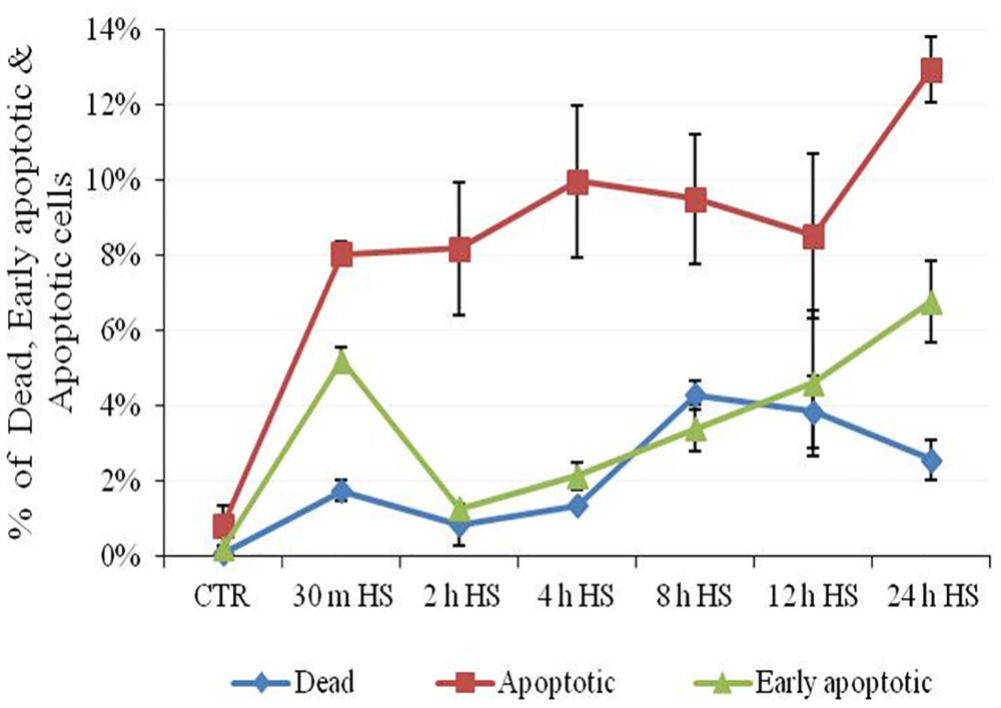
Proportions of early apoptotic, apoptotic and dead cells in unstressed (CTR) and heat stressed treated (TRT) buffalo MECs during recovery period after heat stress.

**Fig 6:**
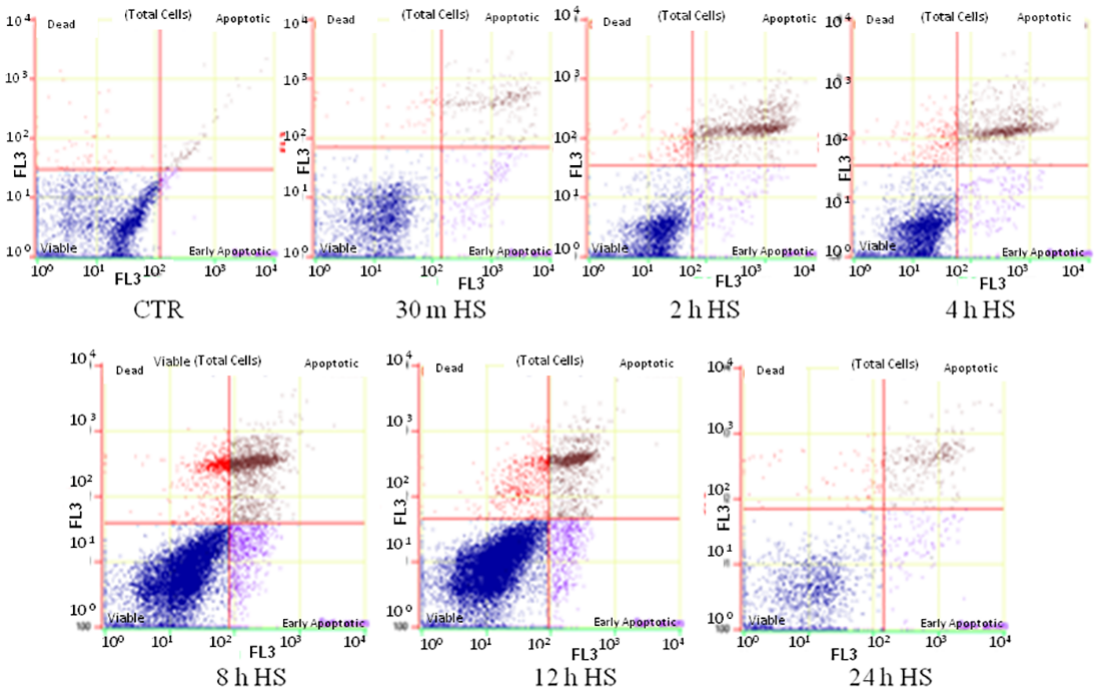
Evaluation of cellular apoptosis based on flow cytometric analysis in unstressed (CTR) and heat stressed (HS) buffalo MECs using Annexin FITC/7-AAD dyes. For each graph, the quadrants display distribution of viable (bottom left), early apoptotic (bottom right), apoptotic (upper right) and dead (upper left) cells.

To check the impact of heat stress on induction of apoptosis or programmed cell death in buffalo MECs, flow cytometric analysis was carried out at various time points of recovery phase post heat stress. The annexinV-FITC /7-AAD dyes were used to detremine the percentage of apoptotic and dead cells. The analysis yielded three different types of cell populations; viable cells with no staining, early apoptotic cells stained positive with annexin-V-FITC dye and late apoptotic/ dead cells stained with both annexin-V-FITC as well as 7-AAD dyes. The cells detected by annexin-V-FITC appearedto be undergoing an early apoptotic process whereas the cells stained with 7-AAD reflected damaged plasma membrane. At 30 min of recovery phase, 5.21% of cells were found to be annexin-V positive suggesting immidiate induction of apoptosis after heat stress. The percentage of MEC undergoing early apoptosis showed progressive increase with increase in time period post heat stress. The percentage of early apoptotic and apoptotic cells was maximum at 24 h post stress with values of 6.79% and 12.95%, respectively. Similarly, the proportion of dead cells also increased with increase in time interval post heat stress (Figs 5 and 6).

Based on present analysis and those reported by other workers [24, 26, 36, and 38] it could be suggested that activation of apoptotic pathway during heat stress could be the major cause of cell death.

### mRNA expression profile of heat shock proteins (HSPs)

The expression pattern of heat shock protein genes (HSPs) showed immediate increase in mRNA level of all the analyzed HSPs in response to *in vitro* heat stress. Each member of the studied molecular chaperons (*HSP27*, *HSP40*, *HSP70*, *HSP60*, and *HSP90*) responded well within 30 min. Most of them showed maximum increase in mRNA expression at 2h-4h,remained elevated till 12h post heat stress and eventually (16h-48h post heat stress) declined to the level of unstressed MECs (S1 Fig). The increase in expression of HSP genes suggested the responsiveness of buffalo MECs to heat stress *in vitro*. The five major heat shock protein genes were selected for analysis as these are the molecular chaperons that ensures the correct protein folding and apoptosis regulation during physiological stressful conditions [39]. Amongst all HSPs, *HSP70* was identified as the most predominant form of transcripts induced in buffalo MECs due to heat stress. The early induction of chaperone activity in buffalo MECs suggested the induction of thermoregulatory mechanism during early stages of cellular response to heat stress. Our findings are in accordance with previous studies conducted across different cell types where it was reported that heat shock remarkably impacts induction of HSP genes; reduction of cellular growth and heat tolerance ability of MECs; and down-regulation of genes associated with cellular metabolism and secretion of milk protein [23,39–43].

#### mRNA expression profile of apoptotic genes

The mRNA expression of two genes related to apoptotic pathway, anti-apoptotic (*BCL2*) and pro-apoptotic (*BAX*) were measured in heat stressed MECs. The results showed that the amount of *BAX* mRNA, which is known as apoptotic activator increased immediately (30m) and continued to increase even till48 h of recovery period after heat stress (S2 Fig). Its expression was highest at 48h time point with 3.439 fold greater followed by 24h time point with 2.25 fold greater than unstressed (control) MECs. Conversely, anti-apoptotic gene (*BCL*2) showed decreased expression up to 24h (0.638 fold) before being recovered close to basal level at 48h (1.037) of heat stress (S2 Fig). The *BAX/BCL2*expression ratio was highest (3.316 fold) after 48h of heat stress, indicating high rate of apoptosis. The expression kinetics of *BAX* and *BCL2* genes strongly indicate the occurrence of heat stress induced apoptosis in the buffalo MECs. The results were in accordance with flow cytometric based apoptotic data showing highest cell death and apoptosis during late time points recovery stages of post heat stress. Several studies have suggested that pro-apoptotic genes like *BAX* accelerates programmed cell death by binding to, and antagonizing the apoptosis repressor *BCL2* gene. This gene interacts with components of permeability transition pore (PTP) protein complex of mitochondria that consists of the voltage-dependent anion channel in the outer mitochondrial membrane and adenine nucleotide translocase in the inner mitochondrial membrane. Under stress conditions, the interaction of *BAX* gene with PTP causes the formation of large pore due to conformational changes resulting in the release of cytochrome *c* and other pro-apoptotic genes that trigger apoptosis. On the other hand, *BCL2* gene prevents the opening of PTP and also encodes an integral outer mitochondrial membrane protein that blocks the apoptotic death. The observed expression profile of *BAX* and *BCL2* genes strongly suggests that heat stress induces cell apoptosis by triggering perturbation of mitochondrial function. Based on our data and information available in the literature, it could be stated that mitochondrial functioning is key to regulate apoptosis and cell death in buffalo MECs during heat stress. During heat stress, pro-apoptotic signals trigger a change in mitochondrial permeability which results in release of mitochondrial proteins into the cytoplasm that might be crucial for apoptosis. In line with our study, few others have also shown induction of cellular apoptosis in different cell lines on heat shock treatment [24, 44].

### Analysis of microarray data for identifying differentially expressed genes

In this study, an attempt was made to obtain a global picture of *in vitro* heat stress response by investigating transcriptome profile of mammary epithelial cells of buffaloes. The Agilent 44K bovine oligonucleotide array which contain ~20,000 probe sets was employed to characterize the gene expression changes in buffalo MEC in response to heat stress. RNA electropherogram profile using bio analyzer (S3 and S4 Figs) revealed that all samples are of good quality and intact. To ensure success in microarray hybridization, the yield and specific activity of Cy3-labeled complementary RNA (cRNA) was measured in terms of cyanine 3 dye concentration (pmol/μL), RNA absorbance ratio (260 nm/280 nm) and cRNA concentration (ng/μL). The concentration of cRNA (ng/μL) was used to determine the μgcRNA yield and concentrations of cRNA (ng/μL) and cyanine 3 (pmol/μL) was used to determine the specific activity In all the MEC samples (CTR, 30 min, 2 h, 4 h, 8 h,1 2h, 16 h and 24 h), the yield of cRNA was >1.65 μg and the specific activity was >9.0 pmol Cy3 per μgcRNA, indicating the good quality of cRNA that was obtained for all the samples.

Out of 40,000 probes in Agilent bovine genome array, we obtained a total of 24573 which passed the expression filter as per the criteria mentioned. Box whisker plot showing distribution of normalized intensity values is presented in S5 Fig. In order to identify differentially expressed genes (DEG), fold change values were generated by subtracting the intensity of unstressed cells (CTR) from those at 30 min, 2 h, 4 h, 8 h, 12 h, 16 h, 24 h heat-treated cells and selected the genes showing at least 3-fold up- or down- regulation at all-time points of heat treatment. The line plot showing transcriptional pattern of DEG filtered at 3-fold cutoff criteria is depicted in S6 Fig. In comparison to unstressed (CTR), the line plot showed lot of variation in transcript pattern during the early time points (30 min, 2 h, 4 h) post heat stress.

**Fig 7:**
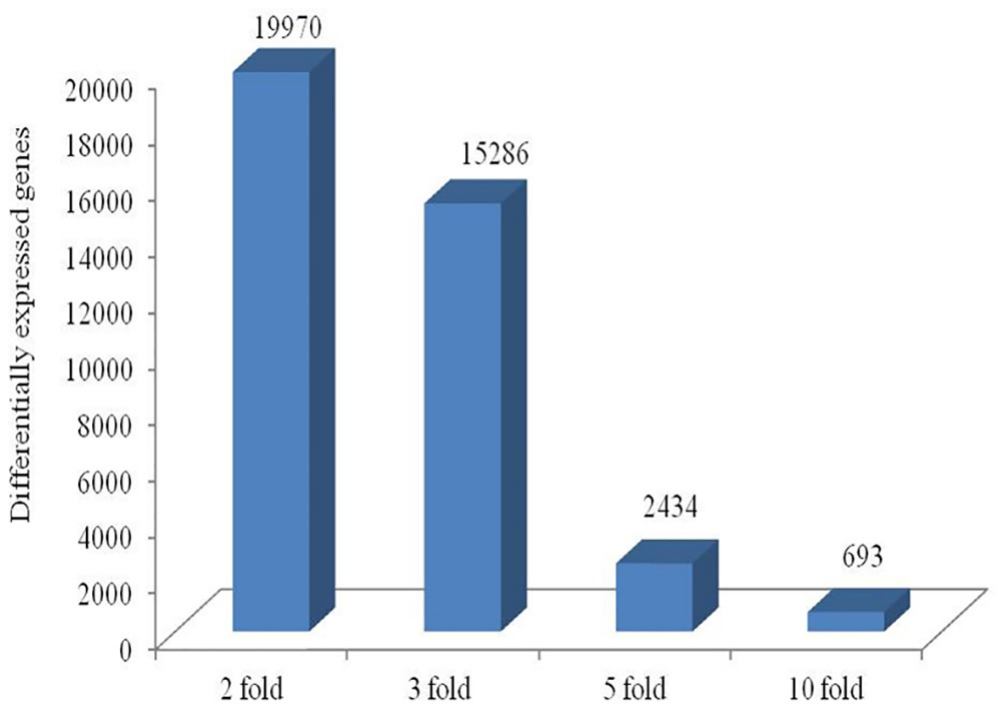
Bar graph showing differentially expressed genes after heat stress in buffalo MECs at different fold change (2, 3, 5 and 10).

**Fig 8:**
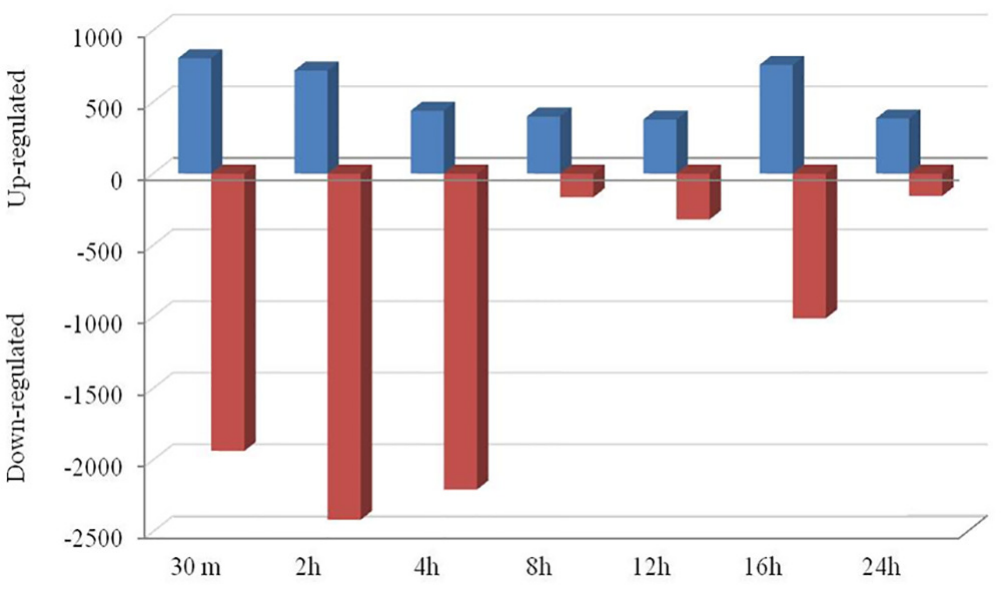
Bar graph showing up- and down-regulated genes at each time point (Fold change 3) in heat stressed buffalo MECs.

The fold change data waslog_2_ transformed and selected for further data analysis. At the cutoff criteria of signed fold change ≥2-or ≤2, a total of 19970 differentially expressed transcripts between unstressed and stressed (all studied time points post stress: 30 min, 2h, 4h, 8h, 12h, 16h, 24h) MECs. At the cutoff criteria of signed fold change ≥3-or ≤3, a total of 15286 DEG was observed across different time points post heat stress. The number of DEG at different fold change criteria (2, 3, 5, 10-fold) is presented in Fig 7. Pair wise comparison with CTR at 3 fold change revealed; 2741 DEG (805/1936 induced/repressed) at 30 min, 3137 DEG (720/2417) at 2h, 2645 DEG at 4h (439/2206), 564 DEG at 8h (399/165), 697 DEG at 12h (377/320), 1769 DEG at 16h (759/1010) and 541 DEG at 24h (385/156) (Fig 8).

**Fig 9:**
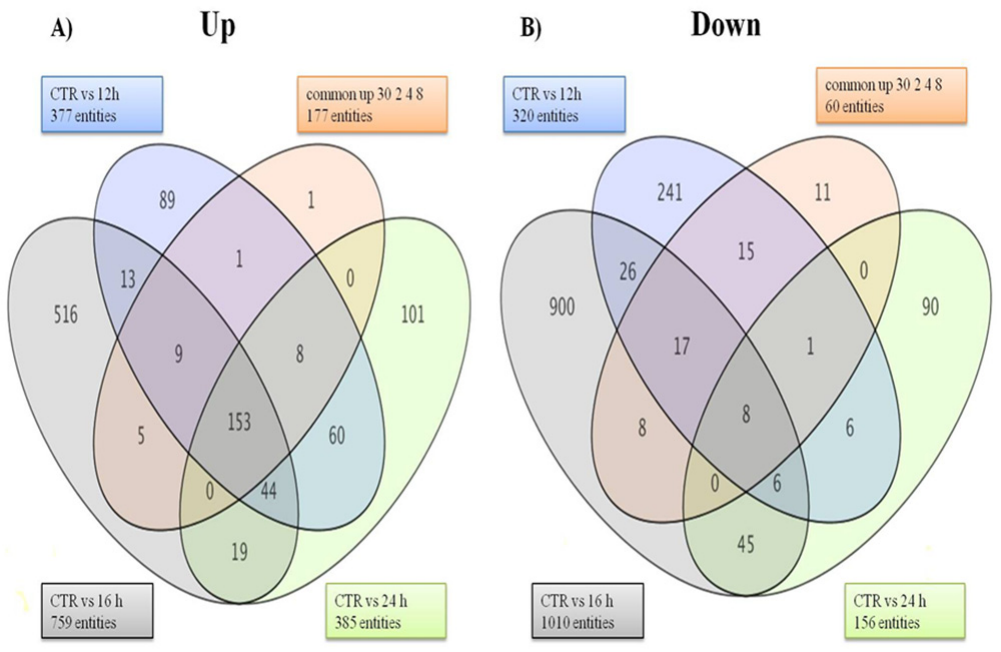
Venn diagrams showing the distribution of genes identified as heat stress responsive at 3 fold change, and the overlapping genes identified as most commonly expressed at all-time points of heat stress treatment in buffalo MECs, (A) most commonly up-regulated (153 genes), (B) most commonly down-regulated (8 genes) at all-time points post heat stress.

**Fig 10:**
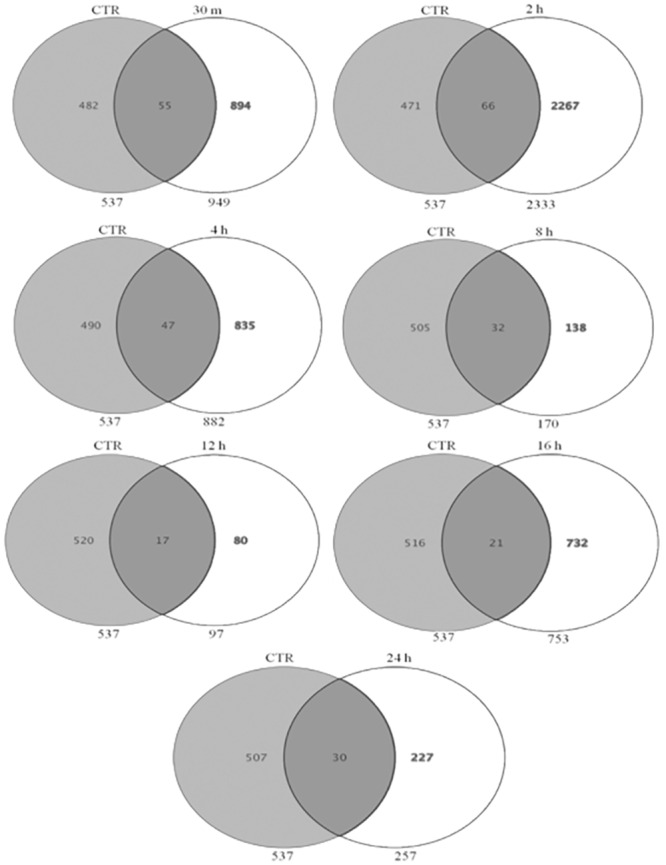
Venn diagram showing number of DEG at 30 min, 2 h, 4 h, 8 h, 12 h, 16 h, and 24 h with respect to unstressed (CTR) at fold change > = 3.0.

**Fig 11:**
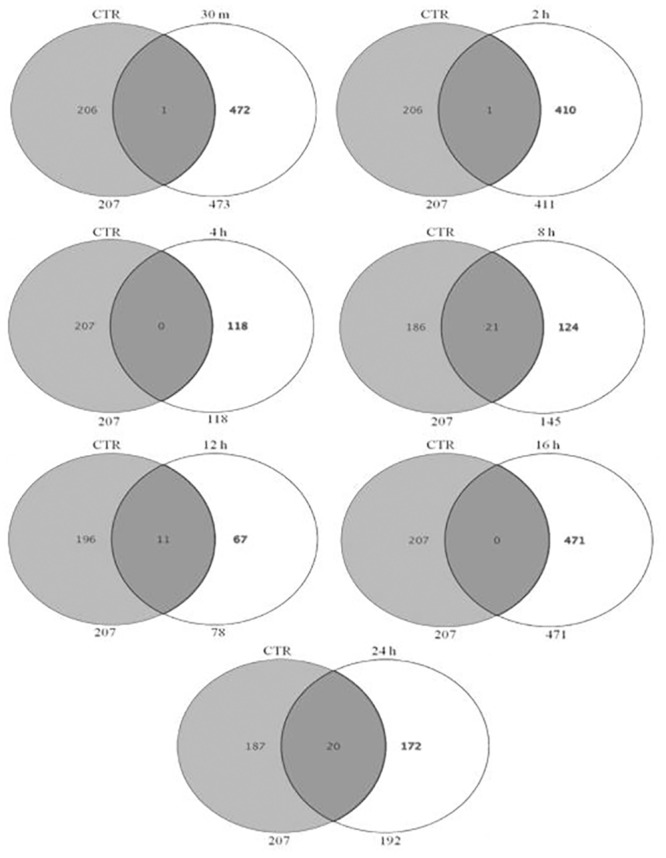
Venn diagram showing number of genes induced at 30 min, 2 h, 4 h, 8 h, 12 h, 16 h, and 24 h with respect to unstressed (CTR) at fold change > = 3.0.

In addition, the Venn analysis showed distribution of genes at 3 fold change and identified list of DEG that were commonly upregulated (153) and down regulated (8) across all time points post heat stress (Fig 9). These genes whose transcriptional pattern changed due to heat stress across all time points were termed as heat responsive genes. The Venn diagram analysis helped to identify the genes that are differentially expressed or remained commonly expressed in unstressed (CTR) and heat stressed MEC at individual time points after heat stress. The analysis revealed that there was relatively a greater transcriptional response during the early time points as evident by 894, 2267, 835 DEG at 30 min, 2h, 4h respectively in comparison to 138, 80, 732 and 227 DEG during late hours i.e. 8h, 12h, 16h and 24h (Fig 10). Further, the Venn analysis was also carried out to find out number of genes that were induced specifically during early and late time points post heat stress in comparison to CTR. During early time points *viz*. 30 min, 2h and 4h, a total of 472, 416 and 118 genes were up regulated respectively in comparison to unstressed (CTR) cells (Fig 11). At later time points *viz*. 8h, 12h, 16h and 24h, a total of 124, 67, 471 and 172 genes were induced in comparison to unstressed cells.

The top 50 up-regulated and top 50 down-regulated transcripts filtered at 3-fold cut off are listed in Table 1 and Table 2, respectively.

**Table 1:** List of top 50 genes up-regulated in heat stressed buffalo MECs (Fold change > = 3.0).

			Fold change > = 3.0 (Relative to control)							
S.No.	Gene_ID	GeneSymbol	30m	2h	4h	8h	12h	16h	24h	Description
1	A_73_P108026	BOLA	8.503	8.157	5.418	5.151	5.126	6.863	5.343	MHC class I heavy chain
2	A_73_P046761	MRPL55	7.623	7.325	7.567	7.213	6.975	7.573	7.242	mitochondrial ribosomal protein L55
3	A_73_118246	PFKFB3	7.231	6.506	3.138	2.763	2.697	4.123	2.861	6-phosphofructo-2-kinase/fructose-2,6-biphosphatase 3
4	A_73_115573	PSMC2	6.850	6.499	6.759	6.945	6.670	6.487	6.767	proteasome (prosome, macropain) 26S subunit, ATPase, 2
5	A_73_118860	ENDOD1	6.156	5.320	5.808	5.828	5.418	6.397	5.988	endonuclease domain containing 1
6	A_73_P030366	ARID5A	6.093	5.457	3.085	3.198	3.136	3.609	3.246	AT rich interactive domain 5A (MRF1-like)
7	A_73_107649	HBXIP	5.747	5.238	4.405	4.628	4.357	4.158	4.325	hepatitis B virus x interacting protein
8	A_73_105502	SENP3	5.182	5.123	5.028	4.684	4.480	4.812	4.548	SUMO1/sentrin/SMT3 specific peptidase 3
9	A_73_P046036	PIM1	4.940	5.002	4.043	3.629	3.351	3.923	3.976	pim-1 oncogene
10	A_73_P046636	TAX1BP3	4.892	4.737	4.906	5.161	5.353	5.225	5.545	Tax1 (human T-cell leukemia virus type I) binding protein 3
11	A_73_P324666	HSPB8	4.451	4.825	4.994	5.311	2.946	4.265	1.281	heat shock 22kDa protein 8
12	A_73_P041676	ATP6V1H	4.440	3.846	3.855	4.248	4.467	4.079	4.597	ATPase, H+ transporting, lysosomal 50/57kDa, V1 subunit H
13	A_73_102559	GDI1	4.427	4.031	3.974	2.945	3.437	4.001	4.085	GDP dissociation inhibitor 1
14	A_73_P421171	C29H11orf68	4.322	4.090	4.481	4.703	4.721	4.610	4.351	chromosome 29 open reading frame, human C11orf68
15	A_73_P334111	NAGA	4.252	3.422	2.778	3.199	2.989	2.996	3.279	N-acetylgalactosaminidase, alpha-
16	A_73_P370971	NCAPH2	4.126	3.587	3.700	3.123	3.623	3.595	3.993	non-SMC condensin II complex, subunit H2
17	A_73_P051671	FAM104A	4.045	3.852	3.630	4.018	3.967	3.489	3.572	family with sequence similarity 104, member A
18	A_73_108441	HRSP12	4.006	3.732	1.963	2.461	2.214	2.333	2.194	heat-responsive protein 12
19	A_73_P394126	CSRNP1	3.995	4.445	1.252	1.733	1.822	1.616	1.314	cysteine-serine-rich nuclear protein 1
20	A_73_121151	SACM1L	3.968	3.250	3.338	3.232	3.232	3.234	3.238	SAC1 suppressor of actin mutations 1-like (yeast)
21	A_73_109582	MTF2	3.903	2.984	3.064	4.861	4.205	2.929	4.034	metal response element binding transcription factor 2
22	A_73_P325281	G3BP1	3.896	3.407	3.788	3.955	3.869	3.619	3.718	GTPase activating protein (SH3 domain) binding protein 1
23	A_73_116684	ARMC6	3.891	3.610	4.225	4.358	4.588	4.281	4.255	armadillo repeat containing 6
24	A_73_P465393	DNAJB2	3.886	3.021	3.424	4.185	4.034	3.189	3.812	DnaJ (Hsp40) homolog, subfamily B, member 2
25	A_73_114308	CDC42EP2	3.807	3.370	3.853	3.629	3.220	4.454	3.028	CDC42 effector protein (Rho GTPase binding) 2
26	A_73_P441761	PMM1	3.774	3.618	4.138	3.406	3.664	3.921	3.817	phosphomannomutase 1
27	A_73_P035201	BRI3	3.734	3.626	3.459	4.010	3.482	3.248	2.986	brain protein I3
28	A_73_P258001	KIAA0020	3.684	3.429	3.501	4.332	3.937	3.381	3.972	KIAA0020
29	A_73_P383651	SQSTM1	3.600	3.672	3.778	4.253	4.391	4.223	3.930	sequestosome 1
30	A_73_P106906	PTPN6	3.580	3.263	1.726	1.969	2.296	1.859	2.212	protein tyrosine phosphatase, non-receptor type 6
31	A_73_108508	CD320	3.565	3.298	4.017	3.296	3.240	3.701	3.206	CD320 molecule
32	A_73_120943	DLGAP4	3.528	2.980	2.628	2.991	3.206	2.823	3.572	discs, large (Drosophila) homolog-associated protein 4
33	A_73_P080981	FAM46A	3.512	3.153	4.728	3.184	2.735	4.460	2.181	family with sequence similarity 46, member A
34	A_73_P510368	SLC26A11	3.336	2.865	2.148	2.487	2.241	2.705	2.251	solute carrier family 26, member 11
35	A_73_100950	IL20RA	3.290	2.940	3.922	2.732	2.767	4.177	3.033	interleukin 20 receptor, alpha
36	A_73_P044846	CNP	3.278	2.988	3.071	2.797	2.828	3.061	2.739	2',3'-cyclic nucleotide 3' phosphodiesterase
37	A_73_107984	TXNDC17	3.266	2.872	3.259	3.667	3.378	3.248	3.421	thioredoxin domain containing 17
38	A_73_104610	TSPO	3.243	2.926	2.940	3.441	3.090	3.670	3.348	translocator protein (18kDa)
39	A_73_101196	RSPRY1	3.234	2.466	1.904	1.903	1.965	1.827	1.874	ring finger and SPRY domain containing 1
40	A_73_106773	KLHDC10	3.224	3.313	3.961	3.515	3.466	3.695	3.042	kelch domain containing 10
41	A_73_102546	SCOC	3.211	3.102	3.385	3.613	3.563	3.260	3.552	short coiled-coil protein
42	A_73_105407	C16H1orf27	3.181	2.925	3.383	3.622	3.585	2.863	3.651	chromosome 16 open reading frame, human C1orf27
43	A_73_P080636	POMT2	3.167	2.657	2.707	2.580	2.336	2.490	2.655	protein-O-mannosyltransferase 2
44	A_73_P034946	SOX4	3.154	3.249	3.356	3.831	4.043	3.737	3.875	SRY (sex determining region Y)-box 4
45	A_73_111382	ZBTB48	3.151	2.648	2.856	2.852	3.125	3.065	2.991	zinc finger and BTB domain containing 48
46	A_73_P340001	MRPS12	3.107	2.614	3.130	2.509	2.838	3.089	3.084	mitochondrial ribosomal protein S12
47	A_73_P040866	SEC61A1	3.085	2.690	2.960	3.435	3.237	2.602	2.968	Sec61 alpha 1 subunit (S. cerevisiae)
48	A_73_120765	CNPY4	3.072	2.987	3.456	3.439	3.034	3.346	3.293	canopy 4 homolog (zebrafish)
49	A_73_115031	GPR137	3.004	2.341	2.488	2.817	2.988	3.002	2.516	G protein-coupled receptor 137
50	A_73_P271701	FUS	2.942	2.768	3.031	2.788	2.501	2.463	2.505	fused in sarcoma

**Table 2:** List of top 50 genes down-regulated in heat stressed buffalo MECs (Fold change > = 3.0).

			Fold change > = 3.0 (Relative to control)							
S.No.	Gene_ID	GeneSymbol	30m	2h	4h	8h	12h	16h	24h	Description
1	A_73_P136431	COL4A1	-5.3	-5.07	-3.34	-0.51	-0.58	-2	-0.7	collagen, type IV, alpha 1
2	A_73_P034721	IGFBP5	-5.2	-5.86	-3.24	-0.8	-0.44	-1.9	-0.2	insulin-like growth factor binding protein 5
3	A_73_101940	CABP2	-4.9	-4.06	-4.03	-0.15	-0.95	-3.1	-0.7	calcium binding protein 2
4	A_73_117103	C11H9orf172	-4.7	-4.52	-3.72	0.15	-0.58	-2.7	-1.8	chromosome 11 open reading frame, human C9orf172
5	A_73_P069121	IREB2	-4.7	-0.89	-0.18	-0.14	-0.31	-0.5	-3.3	iron-responsive element binding protein 2
6	A_73_P038916	FBXO22	-4.7	-0.35	0.03	-0.39	-0.04	-0	-1.4	F-box protein 22
7	A_73_P430271	WIPI2	-4.6	-0.95	-0.42	-0.62	-0.88	0.3	-1.5	WD repeat domain, phosphoinositide interacting 2
8	A_73_112071	C25H16orf59	-4.5	0.06	-0.02	-0.65	-0.5	-0.6	-1.1	chromosome 25 open reading frame, human C16orf59
9	A_73_P075026	NCAM1	-4.5	-5.01	-4.32	-0.75	-1.31	-3.2	-0.6	neural cell adhesion molecule 1
10	A_73_P148631	GPR123	-4.4	-3.84	-2.89	-0.12	-0.46	-2.1	-1.8	G protein-coupled receptor 123
11	A_73_114227	KDELC1	-4.4	-1.75	-1.6	-0.73	-1.29	-1.2	-0.6	KDEL (Lys-Asp-Glu-Leu) containing 1
12	A_73_P313581	LAMA4	-4.3	-4.35	-3.91	-1.26	-1.17	-2.9	-1	laminin, alpha 4
13	A_73_109433	OC90	-4.3	-5.01	-4.84	-0.14	-0.5	-3.2	-0.1	otoconin 90
14	A_73_P035036	PRPS2	-4.2	-3.59	-3.78	-1.05	-0.96	-3.2	-0.2	phosphoribosyl pyrophosphate synthetase 2
15	A_73_120102	HCN3	-4.1	-3.15	-2.75	-0.73	-0.53	-1.3	0.5	hyperpolarization activated cyclic nucleotide-gated potassium channel 3
16	A_73_115436	SYNJ1	-4.1	-3.26	-3.28	-0.03	-0.59	-2.6	-0.4	synaptojanin 1
17	A_73_P035866	PNLIPRP2	-4	-3.4	-3.88	-0.76	-0.6	-1.6	-0.6	pancreatic lipase-related protein 2
18	A_73_P065766	WIPF2	-3.9	-4.18	-4.45	-0.62	-1.37	-2.9	-1.8	WAS/WASL interacting protein family, member 2
19	A_73_P133491	GPX8	-3.9	-3.64	-3.11	0.44	-0.2	-2.7	-0.9	glutathione peroxidase 8 (putative)
20	A_73_P112716	MYLK4	-3.8	-2.64	-3.38	-0.79	-0.73	-2.5	-1.4	myosin light chain kinase family, member 4
21	A_73_106971	BTN2A1	-3.8	-3.61	-3.6	-0.42	-0.75	-3.2	-0.4	butyrophilin, subfamily 2, member A1
22	A_73_119938	KCNJ9	-3.8	-4.66	-1.75	-0.89	-0.37	-0.7	-0.2	potassium inwardly-rectifying channel, subfamily J, member 9
23	A_73_P500853	KRT8	-3.8	-2.69	-2.87	-0.33	-0.55	-2.4	-0.5	keratin 8
24	A_73_115779	PTPN5	-3.8	-3.49	-3.24	-1	-1.28	-2.2	-0.7	protein tyrosine phosphatase, non-receptor type 5 (striatum-enriched)
25	A_73_P490288	RNF222	-3.7	-3.12	-3.43	-0.35	-0.63	-2.7	-0.7	ring finger protein 222
26	A_73_P041186	ABCD3	-3.7	-2.61	-1.87	-0.79	-0.69	-3.2	-0.5	ATP-binding cassette, sub-family D (ALD), member 3
27	A_73_P274726	DDAH1	-3.7	-3.03	-2.92	-0.3	-0.7	-3.6	-0.1	dimethylarginine dimethylaminohydrolase 1
28	A_73_P311601	CHODL	-3.7	-4.51	-4.23	-2.85	-2.66	-2.6	-2.3	chondrolectin
29	A_73_113829	GDF7	-3.7	-3.29	-3.42	-1.35	-1.3	-2.6	-0.5	growth differentiation factor 7
30	A_73_119362	AVPR2	-3.6	-3.52	-1.99	-0.63	-0.68	-3.9	-0.1	arginine vasopressin receptor 2
31	A_73_115939	RHCG	-3.6	-3.93	-4.16	-0.13	-0.37	-2.6	-0.7	Rh family, C glycoprotein
32	A_73_117701	C1D	-3.6	-0.78	-0.84	-0.1	-0.23	-0.7	-1.3	C1D nuclear receptor corepressor
33	A_73_P061011	OR8G5	-3.6	-4.4	-4.11	-0.48	-0.63	-2.5	-0.7	olfactory receptor, family 8, subfamily G, member 5
34	A_73_P335211	TAGLN3	-3.6	-3.08	-2.65	-0.36	-0.26	-1.9	0.1	transgelin 3
35	A_73_117273	NPTX1	-3.6	-2.46	-2.84	-0.76	-0.88	-2.8	-0.2	neuronal pentraxin I
36	A_73_118699	NECAB2	-3.5	-2.57	-2.46	-0.26	-0.23	-1.6	-0.3	N-terminal EF-hand calcium binding protein 2
37	A_73_109252	AMH	-3.5	-4.3	-2.73	-0.52	-0.3	-2.6	-0.1	anti-Mullerian hormone
38	A_73_P059456	TMEM72	-3.5	-2.58	-1.35	-0.67	-0.36	-1.1	-0.2	transmembrane protein 72
39	A_73_110805	LRIT2	-3.5	-4.29	-4.02	-0.84	-0.44	-2.4	0.5	leucine-rich repeat, immunoglobulin-like and transmembrane domains 2
40	A_73_108107	CEP68	-3.5	-3.23	-3	-0.25	-0.16	-2.4	0	centrosomal protein 68kDa
41	A_73_108634	EPYC	-3.4	-4.26	-3.98	-0.57	0.03	-2.4	-0.5	epiphycan
42	A_73_P057846	NPRL3	-3.4	-4.24	-3.61	-0.68	-0.39	-2.4	0.3	nitrogen permease regulator-like 3 (S. cerevisiae)
43	A_73_105589	CAMK1G	-3.4	-2.98	-2.96	-0.33	-0.95	-1.4	-0.7	calcium/calmodulin-dependent protein kinase IG
44	A_73_109422	TRIM15	-3.4	-3.15	-3.24	-2.6	-0.94	-1.7	-0.8	tripartite motif containing 15
45	A_73_P094221	SESTD1	-3.4	-2.06	-0.98	-0.59	0.07	-2.4	-0.7	SEC14 and spectrin domains 1
46	A_73_P096266	TNK2	-3.4	-2.49	-1.43	-0.65	-0.12	-0.7	0.2	tyrosine kinase, non-receptor, 2
47	A_73_115094	KRT35	-3.3	-5.15	-3	-0.55	-0.42	-1.7	-0.1	keratin 35
48	A_73_P036796	ITGB6	-3.3	-1.41	-1.45	-2.42	-3.17	-0.8	-0.7	integrin, beta 6
49	A_73_106188	LRIG3	-3.3	-3.4	-2.01	-0.7	-0.44	-1.5	-0.7	leucine-rich repeats and immunoglobulin-like domains 3
50	A_73_112840	FKBP10	-3.3	-4.13	-3.85	0.38	0.94	-2.3	-1.9	FK506 binding protein 10, 65 kDa

Among most up-regulated genes, *BOLA* was highly up-regulated gene during heat stress in buffalo MECs, probably because the presence of MHC class I raises the possibility of cells in autoimmune disease state [45]. Jorge et al [46] have also reported the induction of *BOLA* gene during early logarithmic growth phase of *Escherichia coli* in response to heat stress. Furthermore, a significant fold change increase in the expression of *BOLA* gene after heat stress in biofilm and planktonic stages of growth in *Escherichia coli* has also been reported [47]. The second most up-regulated gene was *MRPL55* (mitochondrial ribosomal protein) that is implicated in protein synthesis within the mitochondrion and cell cycle progression [48]. Similar to our findings, the heat shock to larval stages of *Drosophila* eye indicated over expression of *MRPL55* transcripts [49]. Another most up-regulated gene *PFKFB* is responsible for maintaining the cellular levels of fructose-2, 6-bisphosphate- a key regulator of glycolysis. Earlier also, significant induction of *PFKFB3* mRNA under hypoxic stress was reported in several human and mouse cell lines [50–51]. In addition, *PSMC2* (proteasome 26S subunit, ATPase, 2) also showed induction in expression pattern during post heat stress in buffalo MECs. This gene is known to be involved in the ATP-dependent degradation of ubiquitinated proteins. Further, it has been observed that *PSMC2* gene also plays an important role in cellular growth and proliferation [52]. The up-regulation of proteosome subunit might indicate the immediate response of *PSMC2* during weakening of ubiquitin-proteosome system resulted in accumulation of abnormal proteins that in turn might confer growth and development in buffalo MECs. Additionally, it has been reported for human chronic myelogenous leukemia cell line that *HSP70* is involved with the dissociation and reassociation of the 26S proteasome during adaptation to oxidative stress [53]. These findings can be correlated with the present study where *HSP70* and *PSMC2* were highly up-regulated, explaining the co-relation between both genes during stressful conditions. Another most up regulated gene observed in present study was *ARID5A*thatplays an important role in development, tissue-specific gene expression, and regulation of cell growth [54]. Along with *ARID5A*, *IL6* was also up regulated conferring the findings that *ARID5A* has a role in stabilization of *IL6* mRNA for promotion of inflammatory responses [55].

Among all down-regulated genes, *COL4A1 (*collagen, type IV, alpha 1*)* gene showed high reduction in expression under heat stress. This gene specifically inhibits endothelial cell proliferation and expression of *HIF- 1alpha* and *ERK1/2* and plays a major role in p38 MAPK activation. Down regulation of collagen, the main component of the extracellular matrix hasalso been reported for sea anemones during heat stress [56]. The next highly down-regulated gene was *IGFBP5 (*insulin-like growth factor binding protein 5*)*. It has a role in tissue turnover by reducing the availability of the survival factor IGF-1 as well as increasing extracellular matrix degradation, thereby causing apoptosis and tissue remolding [57]. Similar to our observation, the reduced mRNA expression of *IGFBP5*has been reported in heat stressed cows as well [58]. Another major affected gene in heat stressed buffalo MECs was *CABP2* (calcium binding protein 2), which is a calcium binding protein and is an important component of calcium mediated cellular signal transduction [59]. Along with above described major genes, *IREB2* (iron-responsive element binding protein 2) also ranked higher among down-regulated genes. The binding of *IREB2* to transferrin receptor mRNA inhibits the degradation of otherwise rapidly degraded mRNA. Its reduced expression was observed in plants during oxidative stress [60] which could be correlated with our study. In addition, *FBXO22* gene, a F-box protein, constitute one of the four subunits of the ubiquitin protein ligase complex called SCFs (SKP1-cullin-F-box), which function in phosphorylation-dependent ubiquitination and are thought to be involved in degradation of specific proteins in response to p53 induction. Similar to our observations, the evolutionarily conserved *Arabidopsis thaliana* F-box protein showed reduction in transcriptional expression profile during temperature stress [61]. Additionally, *NCAM1* gene also showed a reduction pattern in expression level during post heat stress. It is involved in cell-to-cell interactions as well as cell-matrix interactions during development and differentiation and play an important role in immune surveillance. Our findings were in accordance with the reduced expression of *NCAM1* gene in adult mice as a consequence of heat stress [62].

#### Identification of heat stress responsive genes from transcriptome data

Amongst the several hundred genes induced or repressed due to heat stress *in vitro*, an effort was made to filter out genes associated with; heat shock protein family, apoptosis; immune and oxidative stress (Table 3). The heat map view for list of genes identified under these categories is presented in Fig 9. As expected, the whole set of genes of heat shock family *viz*., *HSPA6*, *HSPB8*, *DNAJB2*, *HSPA1A* etc. were up-regulated in MEC at most of the time points post heat stress. The expression of these genes was more during the early phase of heat stress as compared to late time points post heat stress. Our findings are in accordance with previous studies where heat stress led to induction of HSP genes, and down-regulation of genes associated with cellular metabolism and cellular growth [23, 39–40]. Similar to our study, induction in HSPs expression in different cell/tissue types *viz*., leukocytes/lymphocytes [41, 43, 63], bovine endometrial tissue [64, 65], bovine concept uses [65], bovine MECs [23], buffalo lymphocytes [66]due to heat stress has also been reported. Heat stress has also been shown to trigger an increase in *HSP*s in virtually all the vertebrates, including mice [67, 68] domestic goats [69], humans [70, 71], juvenile Hamadryas baboons [72], common carp [73], domestic chickens [74–77] and domestic turkey [78]. Our observations thus supported the idea that *HSP70* can act as reliable, sensitive biomarker of thermal stress [72, 79]. Similarly, several apoptosis related genes were also found to be up-regulated on heat stress. Up-regulation of apoptotic genes could result in disruption of mitochondrion transmembrane potential, thereby causing cytochrome c release leading to the induction in apoptosis. The data on induced expression of apoptotic genes immediately after heat stress suggests that cellular mechanism may not provide protection to the MECs against heat-induced apoptosis in buffalo while during recovery period of heat stress they probably helped the cell to cope with hyperthermia through clearance of damaged proteins. Our findings are in accordance with some previous reports where heat shock showed induction in expression of apoptotic genes in *in vitro* culture models of buffalo embryos [80] and cat skin fibroblasts [81]. In addition, immune system related genes *BOLA*, *IL1-B*, *BOLA-DRA*, *TNF*, *IL1A*, *IL10*, *and CXCL2* and *IL6* were also upregulated by heat shock, supporting the RT-qPCR analysis carried out in the present study. Similar to our findings, the induction of immune related gene expression in intestinal mucosa of mice was observed in response to environmental stress [82]. Furthermore, our findings are supported by increase in expression of *IL6* mRNA in mouse macrophages and MEF cells after hyperthermia [83]. It has also been examined that the exposure of heat stress to mice hepatocytes resulted in *TNFα* induced apoptosis [84]. Along with these findings, effect of heat stress in mammary tissue and peripheral blood mononuclear cells during the dry period in cows revealed altered expression pattern of cytokines exposed to heat stress [85]. Consistent with our study, *CXCL2* expression showed an increase in response to high ambient temperatures in bovine [86–87]. Our observations support the idea of induced expression of pro-inflammatory transcripts (*IL6*, *IL8*, *CXCL2*) in porcine intestinal epithelial cells exposed to infections [88]. Abee and Wouters [89] have also cited that stress adaptive genes such as *BOLA* play a role in controlling cell morphology during heat stress. Our data also showed correlation with increased expression of MHC class genes in porcine intestine [90] and human intestine epithelial cell line [91] during heat stress. In addition, *IL1B* was found to be most induced during inflammatory response in pigs [92]. Likewise, as reported for heat stressed human skin fibroblasts [93],several genes associated with oxidative stress viz., *GSR*, *DUSP16*, *GPX7*, HMOX1, *TXNRD1*, *GPX4* were specifically up-regulated during the early phase of heat stress response in buffalo MECs. Similar to our results, genes of glutathione peroxidase family were shown to be induced under heat stress condition in *Saccharomyces cerevisiae* [94] and quail [95]. The family of GPX is known to play an important role in protecting animals and humans against oxidative stress. In addition the elevated expression of *HMOX1* and *TXNRD1* genes were observed in human melanoma cell culture which confirmed the induction of cellular oxidative stress during harmful insults [96]. Our findings has provided the evidence to suggest the varied expression profile of immune-responsive and oxidative stress related genes in buffalo MECs during heat stress. Thus, in the present study, RT-qPCR analysis validated the transcriptional expression profile of HSPs, apoptotic genes, immune responsive and oxidative stress related genes as observed by microarray gene expression analysis.

**Table 3:** List of genes classified in major functional categories during post heat stress (relative to control) in buffalo MECs.

Heat shock protein family		Fold change > = 3.0 (Relative to control)							
Gene_ID	GeneSymbol	30 m	2 h	4 h	8 h	12 h	16h	24 h	Description
A_73_P092016	HSPA6	5.08	1.57	-1.52	6.14	2.90	0.07	0.43	heat shock 70kDa protein 6 (HSP70B')
A_73_P324666	HSPB8	4.45	4.83	4.99	5.31	2.95	4.27	1.28	heat shock 22kDa protein 8
A_73_P465393	DNAJB2	3.89	3.02	3.42	4.18	4.03	3.19	3.81	DnaJ (Hsp40) homolog, subfamily B, member 2
A_73_P474283	HSPA1A	3.21	2.64	1.54	4.80	2.84	0.41	-1.35	heat shock 70kDa protein 1A
A_73_P262981	DNAJB1	1.60	0.12	0.47	1.73	-0.07	0.25	-0.65	DnaJ (Hsp40) homolog, subfamily B, member 1
A_73_110555	HSPH1	1.49	1.34	0.73	2.86	1.03	-1.24	-0.01	heat shock 105kDa/110kDa protein 1
A_73_P038581	HSPA5	1.39	0.84	1.15	1.75	0.44	-0.15	-1.43	heat shock 70kDa protein 5 (glucose-regulated protein, 78kDa)
**Apoptotic gene family**									
A_73_P108956	BCL2	3.32	2.88	-0.58	-2.11	-2.57	1.26	-0.69	B-cell CLL/lymphoma 2
A_73_100589	IRF5	3.00	2.50	0.25	0.16	0.21	0.86	0.19	interferon regulatory factor 5
A_73_P033666	BCL2L14	2.53	2.24	-0.05	-0.19	-0.79	0.89	0.43	BCL2-like 14 (apoptosis facilitator)
A_73_120639	BCL2L11	2.39	1.96	-0.05	-0.03	0.48	-0.60	0.06	BCL2-like 11 (apoptosis facilitator)
A_73_P087306	NFKB1	2.22	2.59	-0.58	-0.47	-0.15	-0.98	-0.11	nuclear factor of kappa light polypeptide gene enhancer in B-cells 1
A_73_P047636	NFKBIE	2.18	2.04	-0.70	-0.44	-0.18	-0.20	-0.22	nuclear factor of kappa light polypeptide gene enhancer in B-cells inhibitor, epsilon
A_73_105509	PIK3R1	1.68	0.58	0.03	-0.19	0.14	0.68	0.43	phosphoinositide-3-kinase, regulatory subunit 1 (alpha)
A_73_P059646	IRF2	1.65	1.64	1.01	-0.57	-0.42	-1.25	-0.89	interferon regulatory factor 2
A_73_P271841	MCL1	1.33	1.47	-0.67	-0.46	-0.47	-0.27	-0.73	myeloid cell leukemia sequence 1 (BCL2-related)
A_73_104047	CFLAR	1.11	1.85	-1.52	-0.19	0.04	0.07	0.43	CASP8 and FADD-like apoptosis regulator
**Immune system genes**									
A_73_P108026	BOLA	8.50	8.16	5.42	5.15	5.13	6.86	5.34	MHC class I heavy chain
A_73_110556	IL1B	8.33	7.56	-1.30	0.34	0.01	0.12	0.64	interleukin 1, beta
A_73_P038356	BOLA-DRA	7.62	6.75	3.38	7.57	-0.66	5.14	0.09	major histocompatibility complex, class II, DR alpha
A_73_P048896	TNF	7.58	7.47	-1.52	-0.19	-0.79	0.07	0.43	tumor necrosis factor
A_73_P108101	IL1A	7.36	6.88	0.45	-0.19	-0.72	0.07	0.43	interleukin 1, alpha
A_73_P031501	CXCL2	4.15	3.66	0.97	-0.19	0.96	0.07	0.43	chemokine (C-X-C motif) ligand 2
A_73_P030396	IL10	3.44	1.99	-1.82	0.20	0.14	-0.92	0.00	interleukin 10
A_73_P035281	BOLA-N	2.46	3.58	-0.30	-0.35	-0.26	1.44	-0.60	MHC class I antigen
A_73_P087306	NFKB1	2.22	2.59	-0.58	-0.47	-0.15	-0.98	-0.11	nuclear factor of kappa light polypeptide gene enhancer in B-cells 1
A_73_P048171	IL6	2.07	1.49	-1.52	-0.19	-0.79	0.07	0.43	interleukin 6 (interferon, beta 2)
A_73_110221	IL13	-1.05	-1.86	-1.59	0.24	-0.36	0.01	0.37	interleukin 13
A_73_P046791	PDGFA	-1.33	-2.04	-2.54	0.00	-0.19	-3.50	-0.03	platelet-derived growth factor alpha polypeptide
A_73_115863	CD4	-1.39	-2.20	-1.92	-0.21	-0.57	-0.69	-0.20	CD4 molecule
**Oxidative stress related genes**									
A_73_119453	GSR	4.19	3.59	0.47	0.93	0.85	-0.64	0.21	glutathione reductase
A_73_118238	DUSP16	2.02	1.72	-0.01	0.67	-0.85	0.14	0.42	dual specificity phosphatase 16
A_73_103760	GPX7	1.77	1.65	0.00	0.12	0.08	0.67	0.43	glutathione peroxidase 7
A_73_116548	HMOX1	1.74	1.65	1.17	1.66	0.55	2.86	0.26	hemeoxygenase (decycling) 1
A_73_P091051	TXNRD1	1.38	1.39	-0.55	1.23	0.40	-0.52	0.09	thioredoxinreductase 1
A_73_106948	GPX4	1.05	0.57	0.63	-0.19	-0.29	0.03	-0.14	glutathione peroxidase 4
A_73_P296471	SOD3	-1.18	-1.96	-1.72	0.72	0.12	-0.13	1.07	superoxide dismutase 3, extracellular

#### Clustering and annotation of early and late transcriptome data

For creating hierarchical clustering, data of differentially expressed genes across all time points was used. This approach has allowed classifying the whole transcriptome data based on variations in gene expression of heat stressed buffalo MEC at different time points. The analysis generated heat maps to judge for the similarities/patterns between genes and between samples. Based on conditions (time points), the analysis revealed 2 major clusters early time points from 30 min to 4 h post heat stress grouped together while late time points covering 8 h to 24 h along with the control (CTR) formed the second cluster. The clustering data reflected the presence of specificity of expression pattern with respect to time points post heat stress. These results provided evidence that the transcripts were coordinately regulated in a time dependent manner due to heat stress *in vitro*. Hierarchical clustering of differentially expressed genes is depicted in S7 Fig.

The genes from hierarchical clustering were further analyzed to establish the functional groups of differentially expressed genes at different time points post heat stress (early and late time points). For making a functional link and understanding about the biological themes that are enriched in the gene set, Expression Analysis Systematic Explorer (EASE) tool available in Database for Annotation Visualization and Integrated Discovery (DAVID) (*http*:*//david*.*niaid*.*nih*.*gov/david/ease*.*htm*) was used. EASE [97] is generally used as an application tool for rapid biological interpretation of gene lists that could result from the analysis of microarray, proteomics, SAGE and other high-throughput genomic data. We performed functional enrichment analysis against significantly up (151 genes) & down-regulated (628 genes) from first clusters *i*.*e*. early time points (30 min, 2 h and 4 h) and a total of 135 up & 5 down-regulated genes from another cluster of late time points (8 h,12 h, 16 h and 24 h) at fold change 3 with respect to control. Enrichment score (modified fisher exact p-value) for each gene-set calculated in the Gene Ontology terms using DAVID tool [98]. The p-values were computed by a modified Fisher’s exact test for each GO term. We determined the number of genes sharing the same GO terms with a correction for overrepresented (*p* 0.05) categories based on Gene Ontology data. The top most cluster obtained in early up-regulated time points were generally responsible for regulation of cell cycle (6 genes) with highest EASE score (represents more enrichment of the group), apoptosis (6 genes), chaperon activity (7 genes), transcription factor binding (5 genes). The EASE score for GO terms related to up- and down-regulated genes in early and late time points are given in S2 Table. Whereas the cluster in early down-regulated, identified an enrichment of genes associated with signal transduction (27 genes), oxidation reduction (10 genes), response to stimulus (10 genes). Further, highest scoring GOs in late up-regulated include cell cycle (5 genes), negative regulation of apoptosis (4 genes) and late down-regulated genes were primarily related to immune response (15 genes) and cytoskeleton (10 genes). The gene set level analysis revealed various functional groups of gene and related biological mechanism involved in heat stress.

#### Identification of biological process, molecular functions and pathways affected in buffalo MECs during heat stress

In order to explore the biological significance, detailed annotation of gene function, biological process and cellular distribution of differentially expressed genes (DEG; > = 3 fold change) identified in response to heat stress *in vitro* in MEC was accomplished by gene ontology (GO) descriptions. Using the data set of all DEG across all time points post heat stress, a total of 32 biological processes were found to be affected across all time points. However, the four GO terms that was most enriched under biological processes included; response to stimulus (638 genes), multicellular organismal process (506 genes), single organism signaling (381) and cellular developmental process (227 genes. The major five molecular functions identified were binding activity (285), molecular transducer activity (156), receptor activity (143), and transporter activity (25). Under molecular transducer activity, signal transducer was the major molecular function. Signal transducer activity basically conveys a signal across a cell to trigger a response in order to change in cell function or state. Under receptor activity, signal receptor activity (129) was found to be major molecular function associated with DEG. Under transport activity, substrate specific transporter activity (25) and transmembrane transporter activity (25) were affected. The three major cellular processes associated with DEG across all time points were extracellular region (440), membrane (367) and cell part (378).

Additionally, REVIGO analysis was performed on DEG (> = 3 fold change) which summarized major GO terms influenced in buffalo MECs under heat stress. REVIGO is a web server that summarizes long, unintelligible lists of GO terms by finding a representative subset of the terms using a simple clustering algorithm that relies on semantic similarity measures. In the present analysis, a higher frequency of several biological process (cell communication, multicellular organismal development, signal transduction & immune response), cellular component (extracellular region, plasma membrane) & molecular functions (protein binding and transporter activity) were obtained after removing redundant GO terms (Table 4). REVIGO analysis corroborated more or less with the GO analysis that was performed using GO module of GenSpring GX software.

**Table 4:** List of significant GO terms obtained from REVIGO analysis.

Term_ID	Description	frequency	Uniqueness	dispensability	representative
**Biological process**					
GO:0006935	Chemotaxis	2.00%	0.843	0	6935
GO:0016049	cell growth	1.32%	0.895	0.009	16049
GO:0007154	cell communication	27.81%	0.874	0.114	7154
GO:0001666	response to hypoxia	0.61%	0.84	0.114	1666
GO:0007165	signal transduction	25.64%	0.722	0.277	7165
GO:0006810	Transport	17.97%	0.92	0.393	6810
GO:0007275	multicellular organismal development	16.33%	0.836	0.453	7275
GO:0006955	immune response	9.34%	0.829	0.542	6955
GO:0006355	regulation of transcription, DNA-dependent	14.24%	0.73	0.619	6355
**Cellular component**					
GO:0005576	extracellular region	9.77%	0.934	0	5576
GO:0005737	Cytoplasm	42.12%	0.863	0.064	5737
GO:0016021	integral to membrane	29.97%	0.8	0.186	16021
GO:0005886	plasma membrane	23.94%	0.791	0.321	5886
GO:0005739	mitochondrion	9.54%	0.703	0.33	5739
**Molecular function**					
GO:0005215	transporter activity	7.97%	0.957	0	5215
GO:0005515	protein binding	24.46%	0.927	0.034	5515
GO:0004672	protein kinase activity	4.11%	0.886	0.483	4672
GO:0003677	DNA binding	10.35%	0.859	0.486	3677

**Fig 12:**
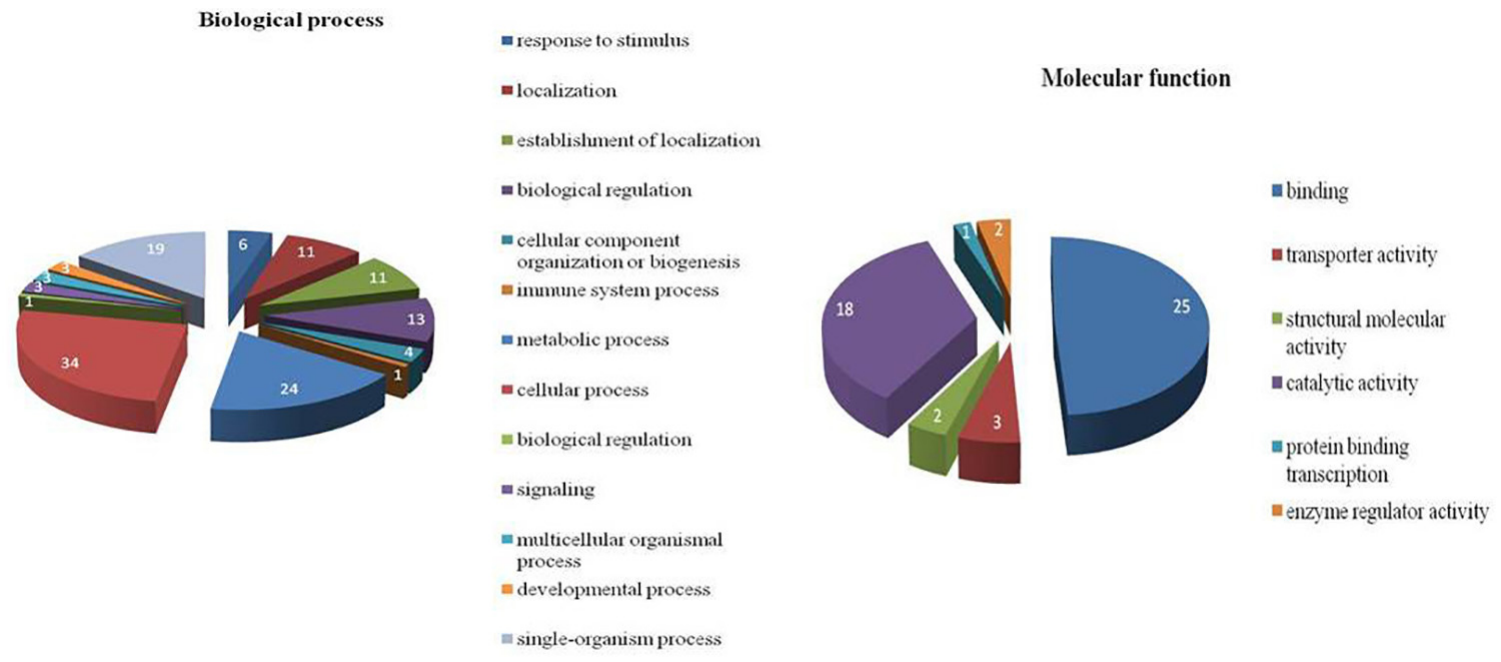
GO categories for biological process enriched across commonly up-regulated 153 genes (relative to control).

Further, 153 genes that were up-regulated in heat stressed MEC across all time points were also assigned with GO terms. A large range of GO categories for biological process were identified including cellular process, metabolic process, single organism process, response to stimulus, biological regulation, immune system processes, signaling *etc*. Among the GO terms associated with response to stimuli in biological processes, the most significant categories were cellular response to stress, response to chemical stimulus and response to stress (Fig 12). GO terms for molecular functions were also identified for 153 genes that were commonly over expressed at all time points (Fig 12). They belong to the categories of catalytic activity (18), binding activity (25), transporter activity (3), structural molecular activity (2) and enzyme regulator activity (2). Binding activity was the second major molecular function activity which was enriched. Various sub-categories like ion binding (13), carbohydrate derivative binding (7), protein binding (11), small molecule binding (9) heterocyclic compound binding (14) etc. were affected.

**Fig 13:**
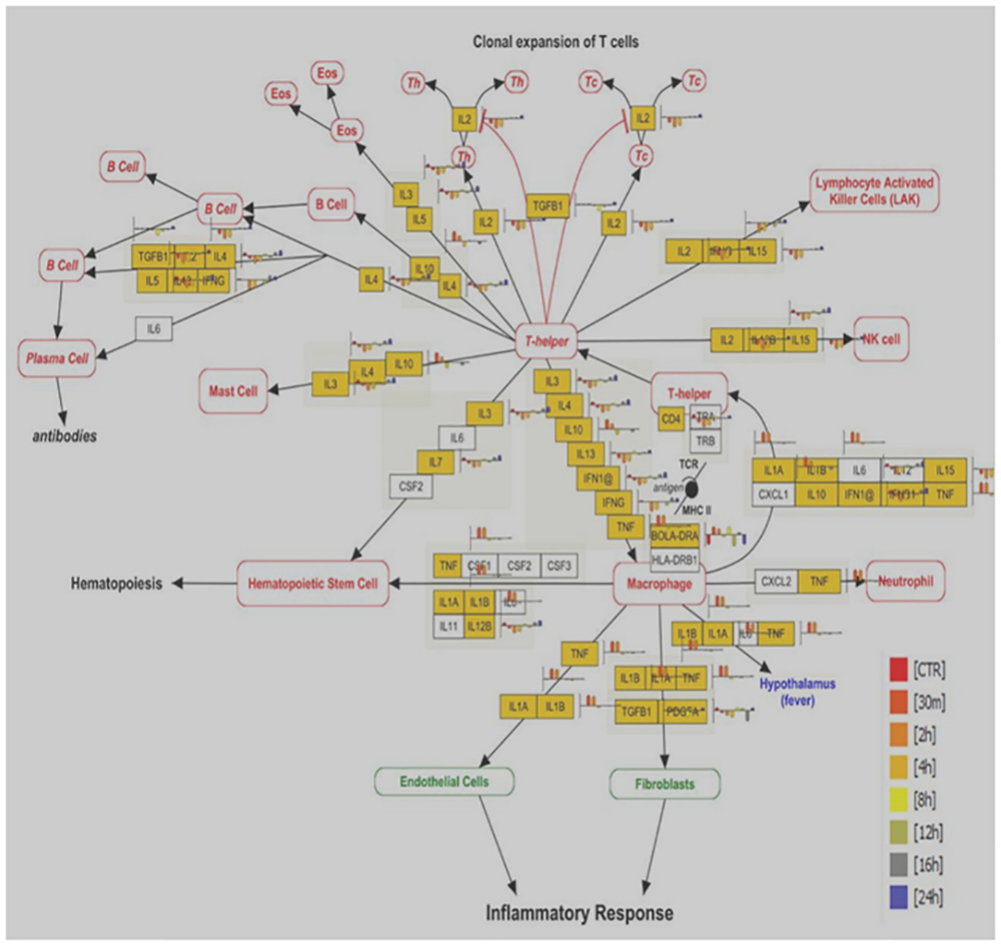
Cytokines & Inflammatory response pathway; shows the significant affected genes (yellow color) across all time points post heat stress.

To get better insight into biological function, we further analyzed the differentially expressed genes based on prior knowledge of biological pathways. Several pathways over represented across all time points were; Electron transport chain, Cytochrome P450 pathway, Apoptosis, IL2 signaling, MAPK, FAS and stress induction of HSP regulation, Delta Notch signaling pathway, Apoptosis modulation by HSP70, EGFR1 signaling, Cytokines and Inflammatory response, Nuclear receptors, Oxidative stress, TNF-alpha/ NF-kB signaling pathway and GPCRs pathway. Representative picture of one of the major signaling pathways; Cytokines & Inflammatory response pathways facilitating cell survival and cell death program is shown in Fig 13.

## Conclusion

The present work thus presented a suitable strategy to characterize the cellular and transcriptomic adaptation of buffalo mammary epithelial cells to heat stress *in-vitro*. Use of heterologous bovine Agilent microarray expression chip in the present study proved successful in dissecting the transcriptome of heat stressed and unstressed buffalo MECs. The study thus has identified several heat responsive genes from different functional classes and biological pathways related to chaperons, immune function, cell proliferation and metabolism etc. known to be affected in by heat stress. The present data provided the strong clue about the coordinated transcriptional response of buffalo mammary epithelial cells to heat stress. The responsiveness of buffalo MECs to heat stress in the present study clearly suggested its suitability as a model to understand the modulation of buffalo mammary gland expression signature in response to environmental heat load. In future, such studies could be extended in evaluating the impact of hyperthermia and other physiological stressors in tissue/cell damage and related gene regulation studies to understand buffalo mammary functions.

## Supporting Information

S1 Fig Expression profile of HSP genes (A) *HSP27*, (B) *HSP40*, (C) *HSP60*, (D) *HSP70* and (E) *HSP90* in buffalo MECs in response to heat stress. (TIF)

S2 Fig mRNA abundance of anti-apoptotic (Bcl2) and pro-apoptotic genes (Bax) in heat stressed buffalo MECs. (TIF)

S3 Fig Virtual gel image of buffalo MEC extracted RNA on bio analyzer (Experion). L: RNA Ladder; CTR: Control; HS: Heat stress
(TIF)

S4 Fig Electropherogram profiles of MEC RNA samples indicating 18S and 28S rRNA peaks. (TIF)

S5 Fig Box whisker plot showing differentially expressed genes (DEGs) after 20-100^th^ percentile normalization showing same median at all time points. (TIF)

S6 Fig Line plot of differentially expressed genes at fold change criteria of ≥3-or ≤3>. (TIF)

S7 Fig Hierarchical clustering across 8 time points with DEGs. The unstressed (CTR) clusters with later stages of heat stress (8 h to 24 h).
(TIF)

S1 Table Candidate target and reference genes evaluated in this study with primer sequences and annealing temperature (Ta). (DOCX)

S2 Table EASE scores for affected GO terms in early and late time points post heat stress. (DOCX)
