# Correction: Impact of Heat Stress on Cellular and Transcriptional Adaptation of Mammary Epithelial Cells in Riverine Buffalo (*Bubalus Bubalis*)

**DOI:** 10.1371/journal.pone.0191380

**Published:** 2018-01-11

**Authors:** Neha Kapila, Ankita Sharma, Amit Kishore, Monika Sodhi, Pawan K. Tripathi, Ashok K. Mohanty, Manishi Mukesh

S1 Table and S2 Table files are incorrect in the list of Supporting Information. Please view the corrected S1 and S2 Tables below.

## Supporting information

S1 Table Candidate target and reference genes evaluated in this study with primer sequences and annealing temperature (Ta). (DOCX)

S2 Table EASE scores for affected GO terms in early and late time points post heat stress. (DOCX)
