# Supplementary figures and images for: Impact of Heat Stress on Cellular and Transcriptional Adaptation of Mammary Epithelial Cells in Riverine Buffalo (Bubalus Bubalis)
Source: PLoS One. 2016 Sep 28;11(9):e0157237. doi: 10.1371/journal.pone.0157237 (PMC5040452; doi:10.1371/journal.pone.0157237)

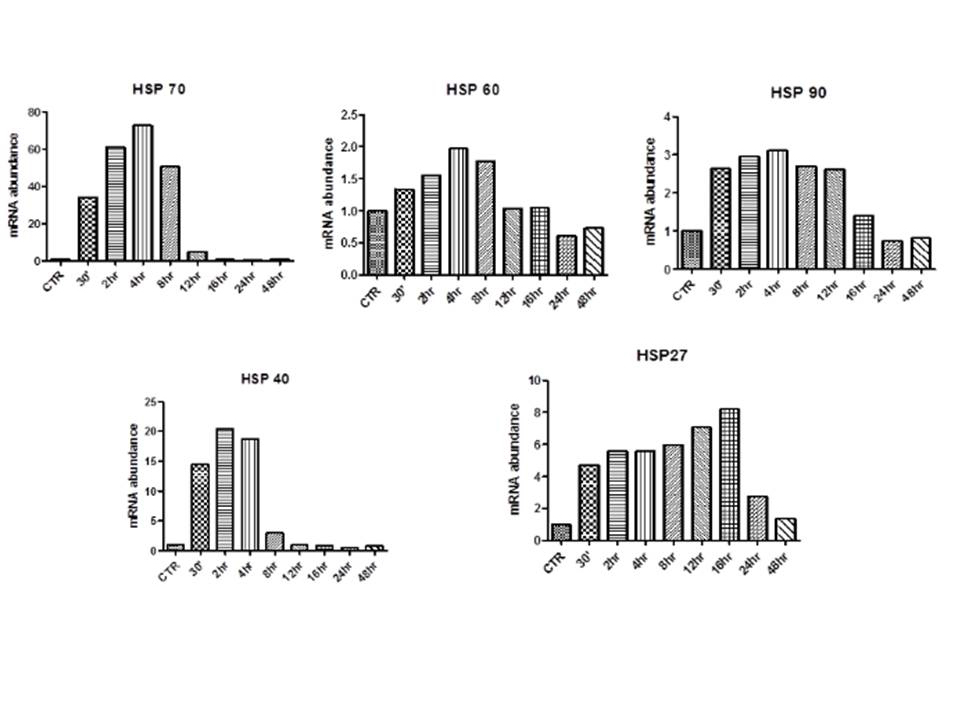

Supplement: S1 Fig — (TIF) [file pone.0157237.s001.tif]

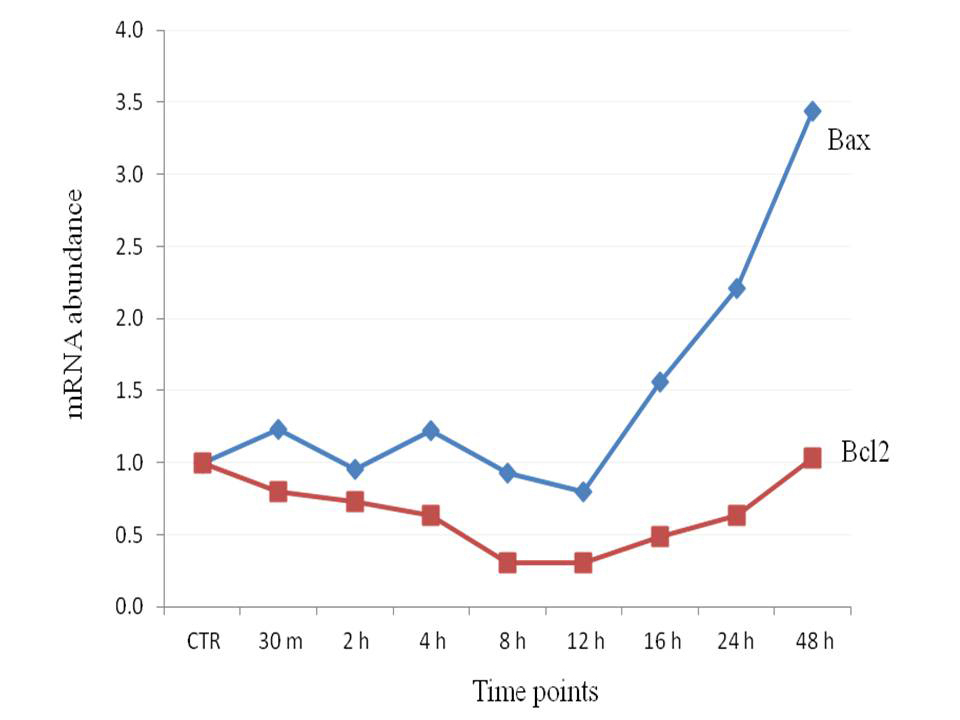

Supplement: S2 Fig — (TIF) [file pone.0157237.s002.tif]

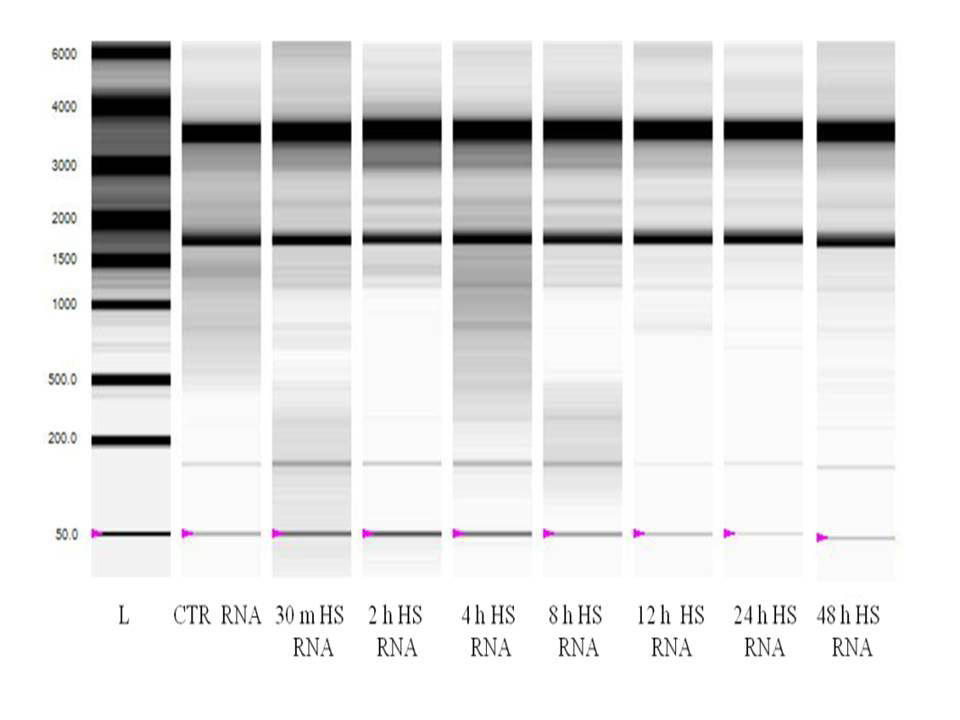

Supplement: S3 Fig — L: RNA Ladder; CTR: Control; HS: Heat stress (TIF) [file pone.0157237.s003.tif]

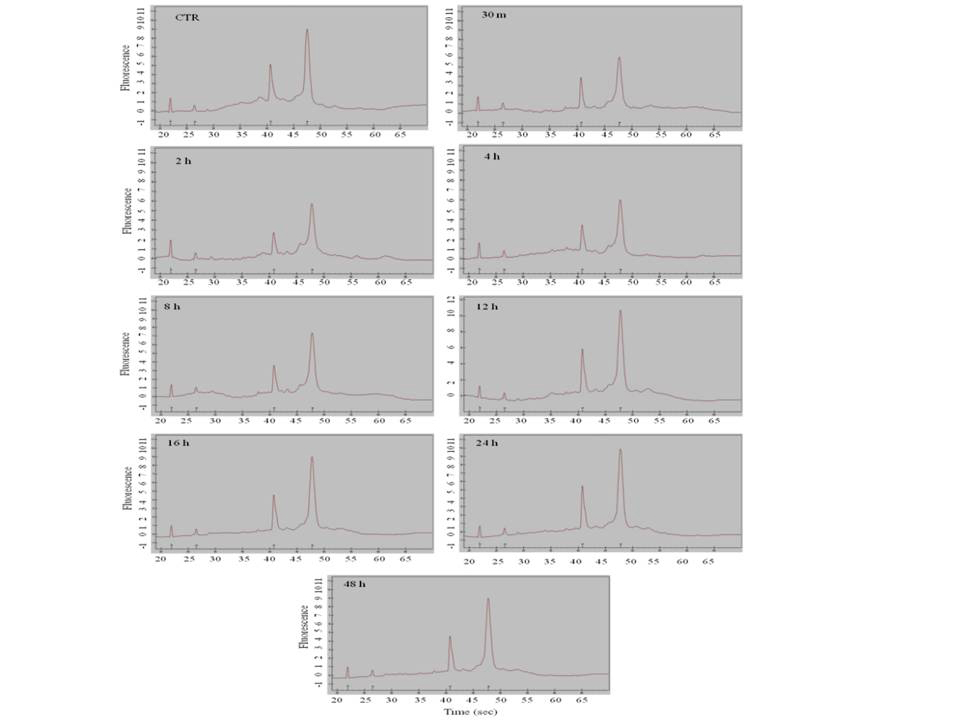

Supplement: S4 Fig — (TIF) [file pone.0157237.s004.tif]

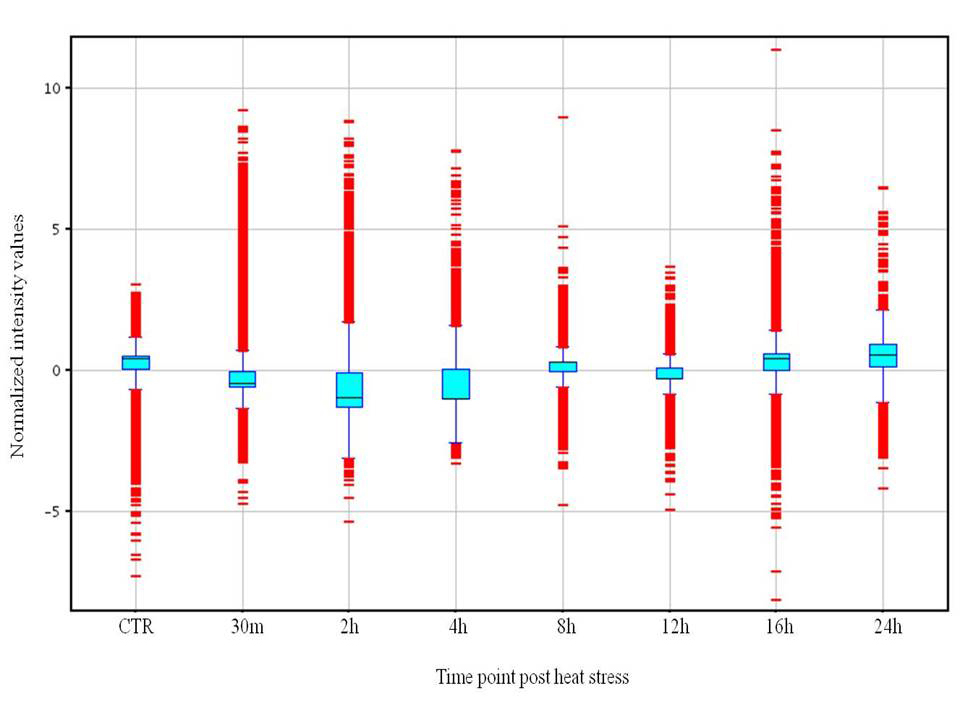

Supplement: S5 Fig — (TIF) [file pone.0157237.s005.tif]

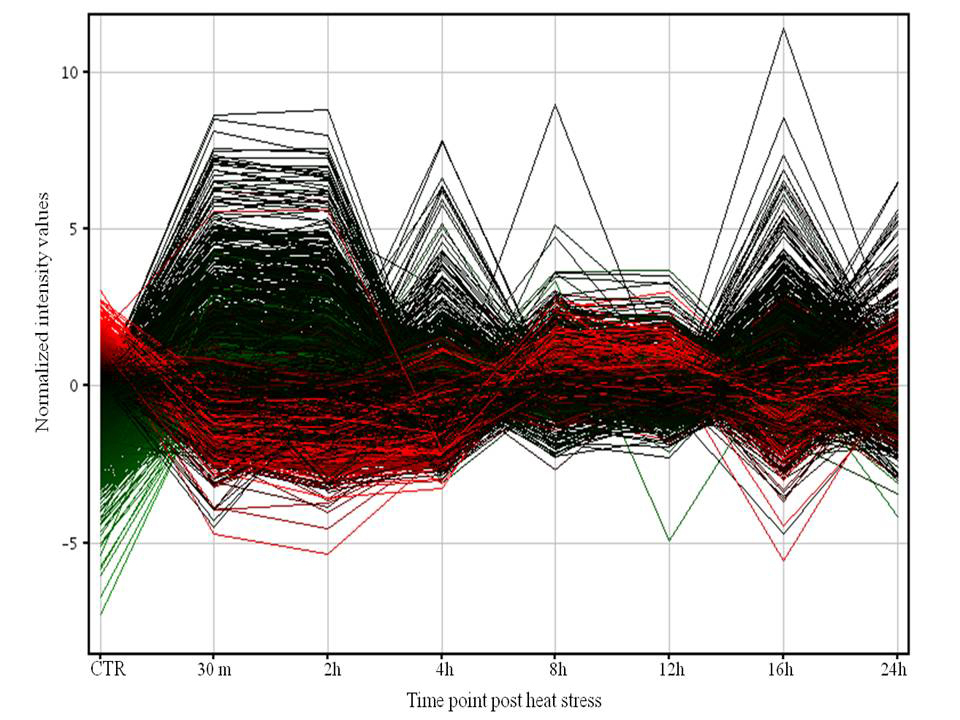

Supplement: S6 Fig — (TIF) [file pone.0157237.s006.tif]

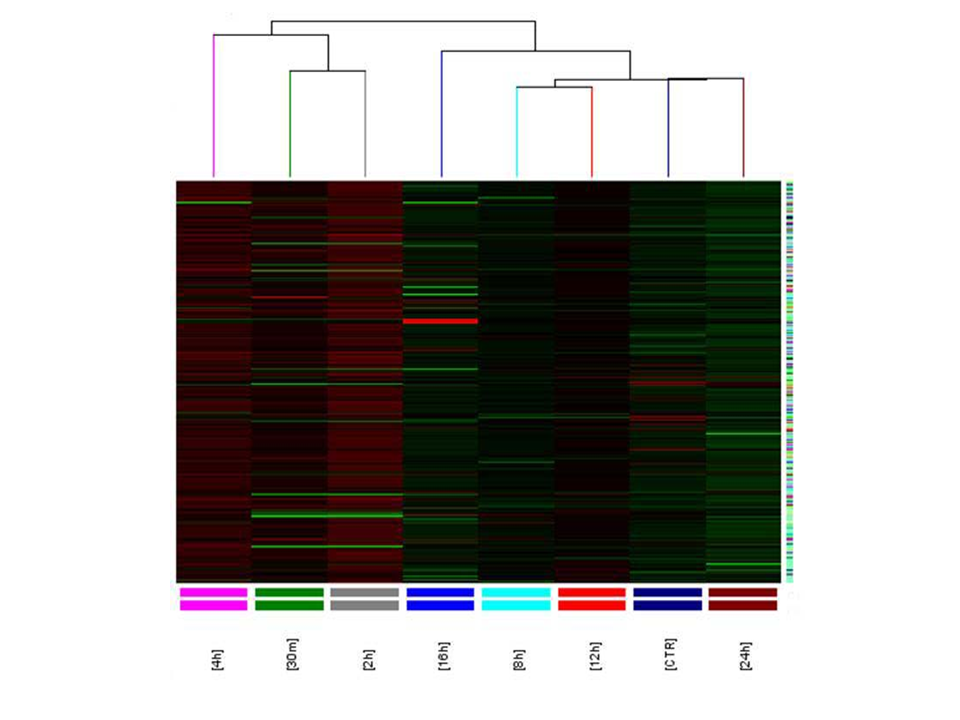

Supplement: S7 Fig — The unstressed (CTR) clusters with later stages of heat stress (8 h to 24 h). (TIF) [file pone.0157237.s007.tif]
