# Supplementary material for: Impact of Heat Stress on Cellular and Transcriptional Adaptation of Mammary Epithelial Cells in Riverine Buffalo (Bubalus Bubalis)
Source: PLoS One. 2016 Sep 28;11(9):e0157237. doi: 10.1371/journal.pone.0157237 (PMC5040452; doi:10.1371/journal.pone.0157237)
Supplement: S1 Table — (DOCX) [file pone.0157237.s008.docx]

**Table 1: List of top 50 genes up-regulated in heat stressed buffalo MECs (Fold change >= 3.0)**

|  |  |  | **Fold change >= 3.0 (Relative to control)** | | | | |  |  |  |
| --- | --- | --- | --- | --- | --- | --- | --- | --- | --- | --- |
| **S.No.** | **Gene_ID** | **GeneSymbol** | **30m** | **2h** | **4h** | **8h** | **12h** | **16h** | **24h** | **Description** |
| 1 | A_73_P108026 | BOLA | 8.503 | 8.157 | 5.418 | 5.151 | 5.126 | 6.863 | 5.343 | MHC class I heavy chain |
| 2 | A_73_P046761 | MRPL55 | 7.623 | 7.325 | 7.567 | 7.213 | 6.975 | 7.573 | 7.242 | mitochondrial ribosomal protein L55 |
| 3 | A_73_118246 | PFKFB3 | 7.231 | 6.506 | 3.138 | 2.763 | 2.697 | 4.123 | 2.861 | 6-phosphofructo-2-kinase/fructose-2,6-biphosphatase 3 |
| 4 | A_73_115573 | PSMC2 | 6.850 | 6.499 | 6.759 | 6.945 | 6.670 | 6.487 | 6.767 | proteasome (prosome, macropain) 26S subunit, ATPase, 2 |
| 5 | A_73_118860 | ENDOD1 | 6.156 | 5.320 | 5.808 | 5.828 | 5.418 | 6.397 | 5.988 | endonuclease domain containing 1 |
| 6 | A_73_P030366 | ARID5A | 6.093 | 5.457 | 3.085 | 3.198 | 3.136 | 3.609 | 3.246 | AT rich interactive domain 5A (MRF1-like) |
| 7 | A_73_107649 | HBXIP | 5.747 | 5.238 | 4.405 | 4.628 | 4.357 | 4.158 | 4.325 | hepatitis B virus x interacting protein |
| 8 | A_73_105502 | SENP3 | 5.182 | 5.123 | 5.028 | 4.684 | 4.480 | 4.812 | 4.548 | SUMO1/sentrin/SMT3 specific peptidase 3 |
| 9 | A_73_P046036 | PIM1 | 4.940 | 5.002 | 4.043 | 3.629 | 3.351 | 3.923 | 3.976 | pim-1 oncogene |
| 10 | A_73_P046636 | TAX1BP3 | 4.892 | 4.737 | 4.906 | 5.161 | 5.353 | 5.225 | 5.545 | Tax1 (human T-cell leukemia virus type I) binding protein 3 |
| 11 | A_73_P324666 | HSPB8 | 4.451 | 4.825 | 4.994 | 5.311 | 2.946 | 4.265 | 1.281 | heat shock 22kDa protein 8 |
| 12 | A_73_P041676 | ATP6V1H | 4.440 | 3.846 | 3.855 | 4.248 | 4.467 | 4.079 | 4.597 | ATPase, H+ transporting, lysosomal 50/57kDa, V1 subunit H |
| 13 | A_73_102559 | GDI1 | 4.427 | 4.031 | 3.974 | 2.945 | 3.437 | 4.001 | 4.085 | GDP dissociation inhibitor 1 |
| 14 | A_73_P421171 | C29H11orf68 | 4.322 | 4.090 | 4.481 | 4.703 | 4.721 | 4.610 | 4.351 | chromosome 29 open reading frame, human C11orf68 |
| 15 | A_73_P334111 | NAGA | 4.252 | 3.422 | 2.778 | 3.199 | 2.989 | 2.996 | 3.279 | N-acetylgalactosaminidase, alpha- |
| 16 | A_73_P370971 | NCAPH2 | 4.126 | 3.587 | 3.700 | 3.123 | 3.623 | 3.595 | 3.993 | non-SMC condensin II complex, subunit H2 |
| 17 | A_73_P051671 | FAM104A | 4.045 | 3.852 | 3.630 | 4.018 | 3.967 | 3.489 | 3.572 | family with sequence similarity 104, member A |
| 18 | A_73_108441 | HRSP12 | 4.006 | 3.732 | 1.963 | 2.461 | 2.214 | 2.333 | 2.194 | heat-responsive protein 12 |
| 19 | A_73_P394126 | CSRNP1 | 3.995 | 4.445 | 1.252 | 1.733 | 1.822 | 1.616 | 1.314 | cysteine-serine-rich nuclear protein 1 |
| 20 | A_73_121151 | SACM1L | 3.968 | 3.250 | 3.338 | 3.232 | 3.232 | 3.234 | 3.238 | SAC1 suppressor of actin mutations 1-like (yeast) |
| 21 | A_73_109582 | MTF2 | 3.903 | 2.984 | 3.064 | 4.861 | 4.205 | 2.929 | 4.034 | metal response element binding transcription factor 2 |
| 22 | A_73_P325281 | G3BP1 | 3.896 | 3.407 | 3.788 | 3.955 | 3.869 | 3.619 | 3.718 | GTPase activating protein (SH3 domain) binding protein 1 |
| 23 | A_73_116684 | ARMC6 | 3.891 | 3.610 | 4.225 | 4.358 | 4.588 | 4.281 | 4.255 | armadillo repeat containing 6 |
| 24 | A_73_P465393 | DNAJB2 | 3.886 | 3.021 | 3.424 | 4.185 | 4.034 | 3.189 | 3.812 | DnaJ (Hsp40) homolog, subfamily B, member 2 |
| 25 | A_73_114308 | CDC42EP2 | 3.807 | 3.370 | 3.853 | 3.629 | 3.220 | 4.454 | 3.028 | CDC42 effector protein (Rho GTPase binding) 2 |
| 26 | A_73_P441761 | PMM1 | 3.774 | 3.618 | 4.138 | 3.406 | 3.664 | 3.921 | 3.817 | phosphomannomutase 1 |
| 27 | A_73_P035201 | BRI3 | 3.734 | 3.626 | 3.459 | 4.010 | 3.482 | 3.248 | 2.986 | brain protein I3 |
| 28 | A_73_P258001 | KIAA0020 | 3.684 | 3.429 | 3.501 | 4.332 | 3.937 | 3.381 | 3.972 | KIAA0020 |
| 29 | A_73_P383651 | SQSTM1 | 3.600 | 3.672 | 3.778 | 4.253 | 4.391 | 4.223 | 3.930 | sequestosome 1 |
| 30 | A_73_P106906 | PTPN6 | 3.580 | 3.263 | 1.726 | 1.969 | 2.296 | 1.859 | 2.212 | protein tyrosine phosphatase, non-receptor type 6 |
| 31 | A_73_108508 | CD320 | 3.565 | 3.298 | 4.017 | 3.296 | 3.240 | 3.701 | 3.206 | CD320 molecule |
| 32 | A_73_120943 | DLGAP4 | 3.528 | 2.980 | 2.628 | 2.991 | 3.206 | 2.823 | 3.572 | discs, large (Drosophila) homolog-associated protein 4 |
| 33 | A_73_P080981 | FAM46A | 3.512 | 3.153 | 4.728 | 3.184 | 2.735 | 4.460 | 2.181 | family with sequence similarity 46, member A |
| 34 | A_73_P510368 | SLC26A11 | 3.336 | 2.865 | 2.148 | 2.487 | 2.241 | 2.705 | 2.251 | solute carrier family 26, member 11 |
| 35 | A_73_100950 | IL20RA | 3.290 | 2.940 | 3.922 | 2.732 | 2.767 | 4.177 | 3.033 | interleukin 20 receptor, alpha |
| 36 | A_73_P044846 | CNP | 3.278 | 2.988 | 3.071 | 2.797 | 2.828 | 3.061 | 2.739 | 2',3'-cyclic nucleotide 3' phosphodiesterase |
| 37 | A_73_107984 | TXNDC17 | 3.266 | 2.872 | 3.259 | 3.667 | 3.378 | 3.248 | 3.421 | thioredoxin domain containing 17 |
| 38 | A_73_104610 | TSPO | 3.243 | 2.926 | 2.940 | 3.441 | 3.090 | 3.670 | 3.348 | translocator protein (18kDa) |
| 39 | A_73_101196 | RSPRY1 | 3.234 | 2.466 | 1.904 | 1.903 | 1.965 | 1.827 | 1.874 | ring finger and SPRY domain containing 1 |
| 40 | A_73_106773 | KLHDC10 | 3.224 | 3.313 | 3.961 | 3.515 | 3.466 | 3.695 | 3.042 | kelch domain containing 10 |
| 41 | A_73_102546 | SCOC | 3.211 | 3.102 | 3.385 | 3.613 | 3.563 | 3.260 | 3.552 | short coiled-coil protein |
| 42 | A_73_105407 | C16H1orf27 | 3.181 | 2.925 | 3.383 | 3.622 | 3.585 | 2.863 | 3.651 | chromosome 16 open reading frame, human C1orf27 |
| 43 | A_73_P080636 | POMT2 | 3.167 | 2.657 | 2.707 | 2.580 | 2.336 | 2.490 | 2.655 | protein-O-mannosyltransferase 2 |
| 44 | A_73_P034946 | SOX4 | 3.154 | 3.249 | 3.356 | 3.831 | 4.043 | 3.737 | 3.875 | SRY (sex determining region Y)-box 4 |
| 45 | A_73_111382 | ZBTB48 | 3.151 | 2.648 | 2.856 | 2.852 | 3.125 | 3.065 | 2.991 | zinc finger and BTB domain containing 48 |
| 46 | A_73_P340001 | MRPS12 | 3.107 | 2.614 | 3.130 | 2.509 | 2.838 | 3.089 | 3.084 | mitochondrial ribosomal protein S12 |
| 47 | A_73_P040866 | SEC61A1 | 3.085 | 2.690 | 2.960 | 3.435 | 3.237 | 2.602 | 2.968 | Sec61 alpha 1 subunit (S. cerevisiae) |
| 48 | A_73_120765 | CNPY4 | 3.072 | 2.987 | 3.456 | 3.439 | 3.034 | 3.346 | 3.293 | canopy 4 homolog (zebrafish) |
| 49 | A_73_115031 | GPR137 | 3.004 | 2.341 | 2.488 | 2.817 | 2.988 | 3.002 | 2.516 | G protein-coupled receptor 137 |
| 50 | A_73_P271701 | FUS | 2.942 | 2.768 | 3.031 | 2.788 | 2.501 | 2.463 | 2.505 | fused in sarcoma |
