# Supplementary material for: Impact of Heat Stress on Cellular and Transcriptional Adaptation of Mammary Epithelial Cells in Riverine Buffalo (Bubalus Bubalis)
Source: PLoS One. 2016 Sep 28;11(9):e0157237. doi: 10.1371/journal.pone.0157237 (PMC5040452; doi:10.1371/journal.pone.0157237)
Supplement: S2 Table — (DOCX) [file pone.0157237.s009.docx]

**Table 2: List of top 50 genes down-regulated in heat stressed buffalo MECs (Fold change >= 3.0)**

|  |  |  | **Fold change >= 3.0 (Relative to control)** | | | | | | |  |
| --- | --- | --- | --- | --- | --- | --- | --- | --- | --- | --- |
| **S.No.** | **Gene_ID** | **GeneSymbol** | **30m** | **2h** | **4h** | **8h** | **12h** | **16h** | **24h** | **Description** |
| 1 | A_73_P136431 | COL4A1 | -5.3 | -5.07 | -3.34 | -0.51 | -0.58 | -2 | -0.7 | collagen, type IV, alpha 1 |
| 2 | A_73_P034721 | IGFBP5 | -5.2 | -5.86 | -3.24 | -0.8 | -0.44 | -1.9 | -0.2 | insulin-like growth factor binding protein 5 |
| 3 | A_73_101940 | CABP2 | -4.9 | -4.06 | -4.03 | -0.15 | -0.95 | -3.1 | -0.7 | calcium binding protein 2 |
| 4 | A_73_117103 | C11H9orf172 | -4.7 | -4.52 | -3.72 | 0.15 | -0.58 | -2.7 | -1.8 | chromosome 11 open reading frame, human C9orf172 |
| 5 | A_73_P069121 | IREB2 | -4.7 | -0.89 | -0.18 | -0.14 | -0.31 | -0.5 | -3.3 | iron-responsive element binding protein 2 |
| 6 | A_73_P038916 | FBXO22 | -4.7 | -0.35 | 0.03 | -0.39 | -0.04 | -0 | -1.4 | F-box protein 22 |
| 7 | A_73_P430271 | WIPI2 | -4.6 | -0.95 | -0.42 | -0.62 | -0.88 | 0.3 | -1.5 | WD repeat domain, phosphoinositide interacting 2 |
| 8 | A_73_112071 | C25H16orf59 | -4.5 | 0.06 | -0.02 | -0.65 | -0.5 | -0.6 | -1.1 | chromosome 25 open reading frame, human C16orf59 |
| 9 | A_73_P075026 | NCAM1 | -4.5 | -5.01 | -4.32 | -0.75 | -1.31 | -3.2 | -0.6 | neural cell adhesion molecule 1 |
| 10 | A_73_P148631 | GPR123 | -4.4 | -3.84 | -2.89 | -0.12 | -0.46 | -2.1 | -1.8 | G protein-coupled receptor 123 |
| 11 | A_73_114227 | KDELC1 | -4.4 | -1.75 | -1.6 | -0.73 | -1.29 | -1.2 | -0.6 | KDEL (Lys-Asp-Glu-Leu) containing 1 |
| 12 | A_73_P313581 | LAMA4 | -4.3 | -4.35 | -3.91 | -1.26 | -1.17 | -2.9 | -1 | laminin, alpha 4 |
| 13 | A_73_109433 | OC90 | -4.3 | -5.01 | -4.84 | -0.14 | -0.5 | -3.2 | -0.1 | otoconin 90 |
| 14 | A_73_P035036 | PRPS2 | -4.2 | -3.59 | -3.78 | -1.05 | -0.96 | -3.2 | -0.2 | phosphoribosyl pyrophosphate synthetase 2 |
| 15 | A_73_120102 | HCN3 | -4.1 | -3.15 | -2.75 | -0.73 | -0.53 | -1.3 | 0.5 | hyperpolarization activated cyclic nucleotide-gated potassium channel 3 |
| 16 | A_73_115436 | SYNJ1 | -4.1 | -3.26 | -3.28 | -0.03 | -0.59 | -2.6 | -0.4 | synaptojanin 1 |
| 17 | A_73_P035866 | PNLIPRP2 | -4 | -3.4 | -3.88 | -0.76 | -0.6 | -1.6 | -0.6 | pancreatic lipase-related protein 2 |
| 18 | A_73_P065766 | WIPF2 | -3.9 | -4.18 | -4.45 | -0.62 | -1.37 | -2.9 | -1.8 | WAS/WASL interacting protein family, member 2 |
| 19 | A_73_P133491 | GPX8 | -3.9 | -3.64 | -3.11 | 0.44 | -0.2 | -2.7 | -0.9 | glutathione peroxidase 8 (putative) |
| 20 | A_73_P112716 | MYLK4 | -3.8 | -2.64 | -3.38 | -0.79 | -0.73 | -2.5 | -1.4 | myosin light chain kinase family, member 4 |
| 21 | A_73_106971 | BTN2A1 | -3.8 | -3.61 | -3.6 | -0.42 | -0.75 | -3.2 | -0.4 | butyrophilin, subfamily 2, member A1 |
| 22 | A_73_119938 | KCNJ9 | -3.8 | -4.66 | -1.75 | -0.89 | -0.37 | -0.7 | -0.2 | potassium inwardly-rectifying channel, subfamily J, member 9 |
| 23 | A_73_P500853 | KRT8 | -3.8 | -2.69 | -2.87 | -0.33 | -0.55 | -2.4 | -0.5 | keratin 8 |
| 24 | A_73_115779 | PTPN5 | -3.8 | -3.49 | -3.24 | -1 | -1.28 | -2.2 | -0.7 | protein tyrosine phosphatase, non-receptor type 5 (striatum-enriched) |
| 25 | A_73_P490288 | RNF222 | -3.7 | -3.12 | -3.43 | -0.35 | -0.63 | -2.7 | -0.7 | ring finger protein 222 |
| 26 | A_73_P041186 | ABCD3 | -3.7 | -2.61 | -1.87 | -0.79 | -0.69 | -3.2 | -0.5 | ATP-binding cassette, sub-family D (ALD), member 3 |
| 27 | A_73_P274726 | DDAH1 | -3.7 | -3.03 | -2.92 | -0.3 | -0.7 | -3.6 | -0.1 | dimethylarginine dimethylaminohydrolase 1 |
| 28 | A_73_P311601 | CHODL | -3.7 | -4.51 | -4.23 | -2.85 | -2.66 | -2.6 | -2.3 | chondrolectin |
| 29 | A_73_113829 | GDF7 | -3.7 | -3.29 | -3.42 | -1.35 | -1.3 | -2.6 | -0.5 | growth differentiation factor 7 |
| 30 | A_73_119362 | AVPR2 | -3.6 | -3.52 | -1.99 | -0.63 | -0.68 | -3.9 | -0.1 | arginine vasopressin receptor 2 |
| 31 | A_73_115939 | RHCG | -3.6 | -3.93 | -4.16 | -0.13 | -0.37 | -2.6 | -0.7 | Rh family, C glycoprotein |
| 32 | A_73_117701 | C1D | -3.6 | -0.78 | -0.84 | -0.1 | -0.23 | -0.7 | -1.3 | C1D nuclear receptor corepressor |
| 33 | A_73_P061011 | OR8G5 | -3.6 | -4.4 | -4.11 | -0.48 | -0.63 | -2.5 | -0.7 | olfactory receptor, family 8, subfamily G, member 5 |
| 34 | A_73_P335211 | TAGLN3 | -3.6 | -3.08 | -2.65 | -0.36 | -0.26 | -1.9 | 0.1 | transgelin 3 |
| 35 | A_73_117273 | NPTX1 | -3.6 | -2.46 | -2.84 | -0.76 | -0.88 | -2.8 | -0.2 | neuronal pentraxin I |
| 36 | A_73_118699 | NECAB2 | -3.5 | -2.57 | -2.46 | -0.26 | -0.23 | -1.6 | -0.3 | N-terminal EF-hand calcium binding protein 2 |
| 37 | A_73_109252 | AMH | -3.5 | -4.3 | -2.73 | -0.52 | -0.3 | -2.6 | -0.1 | anti-Mullerian hormone |
| 38 | A_73_P059456 | TMEM72 | -3.5 | -2.58 | -1.35 | -0.67 | -0.36 | -1.1 | -0.2 | transmembrane protein 72 |
| 39 | A_73_110805 | LRIT2 | -3.5 | -4.29 | -4.02 | -0.84 | -0.44 | -2.4 | 0.5 | leucine-rich repeat, immunoglobulin-like and transmembrane domains 2 |
| 40 | A_73_108107 | CEP68 | -3.5 | -3.23 | -3 | -0.25 | -0.16 | -2.4 | 0 | centrosomal protein 68kDa |
| 41 | A_73_108634 | EPYC | -3.4 | -4.26 | -3.98 | -0.57 | 0.03 | -2.4 | -0.5 | epiphycan |
| 42 | A_73_P057846 | NPRL3 | -3.4 | -4.24 | -3.61 | -0.68 | -0.39 | -2.4 | 0.3 | nitrogen permease regulator-like 3 (S. cerevisiae) |
| 43 | A_73_105589 | CAMK1G | -3.4 | -2.98 | -2.96 | -0.33 | -0.95 | -1.4 | -0.7 | calcium/calmodulin-dependent protein kinase IG |
| 44 | A_73_109422 | TRIM15 | -3.4 | -3.15 | -3.24 | -2.6 | -0.94 | -1.7 | -0.8 | tripartite motif containing 15 |
| 45 | A_73_P094221 | SESTD1 | -3.4 | -2.06 | -0.98 | -0.59 | 0.07 | -2.4 | -0.7 | SEC14 and spectrin domains 1 |
| 46 | A_73_P096266 | TNK2 | -3.4 | -2.49 | -1.43 | -0.65 | -0.12 | -0.7 | 0.2 | tyrosine kinase, non-receptor, 2 |
| 47 | A_73_115094 | KRT35 | -3.3 | -5.15 | -3 | -0.55 | -0.42 | -1.7 | -0.1 | keratin 35 |
| 48 | A_73_P036796 | ITGB6 | -3.3 | -1.41 | -1.45 | -2.42 | -3.17 | -0.8 | -0.7 | integrin, beta 6 |
| 49 | A_73_106188 | LRIG3 | -3.3 | -3.4 | -2.01 | -0.7 | -0.44 | -1.5 | -0.7 | leucine-rich repeats and immunoglobulin-like domains 3 |
| 50 | A_73_112840 | FKBP10 | -3.3 | -4.13 | -3.85 | 0.38 | 0.94 | -2.3 | -1.9 | FK506 binding protein 10, 65 kDa |
