# Supplementary material for: Correction: Impact of Heat Stress on Cellular and Transcriptional Adaptation of Mammary Epithelial Cells in Riverine Buffalo (Bubalus Bubalis)
Source: PLoS One. 2018 Jan 11;13(1):e0191380. doi: 10.1371/journal.pone.0191380 (PMC5764454; doi:10.1371/journal.pone.0191380)
Supplement: S1 Table — (DOCX) [file pone.0191380.s001.docx]

**Supplementary Table 1: Candidate target and reference genes evaluated in this study with primer sequences and annealing temperature (Ta)**

| **Gene Symbol** | **Primers 5'-3' (forward, reverse)** | **Ta (°C)** |
| --- | --- | --- |
| *HSP27* | TACATTTCCCGTTGCTTC; ACGGACAGAGAGGAGGAGAC | 60 |
| *HSP40* | AGCCAGGATCAGCCTTC;  AACACAACGGGTATGGT | 60 |
| *HSP60* | CGACAACTTCTGCTGTTGTTA; ATGATGCTATGCTTGGAGAT | 60 |
| *HSP70* | AACATGAAGAGCGCCGTGGAGG; GTTACACACCTGCTCCAGCTCC | 60 |
| *HSP90* | CTGTCATCAGCAGTGGG;  ACATGCCAACAGGATCTAC | 60 |
| *RPL4* | TTGGAAACATGTGTCGTGGG  GCAGATGGCGTATCGCTTCT | 60 |
| *EEF1A1* | CATCCCAGGCTGACTGTGC  TGTAAGCCAAAAGGGCATGC | 60 |
| *RPS23* | CCCAATGATGGTTGCTTGAA  CGGACTCCAGGAATGTCACC | 60 |
