# Supplementary material for: Correction: Impact of Heat Stress on Cellular and Transcriptional Adaptation of Mammary Epithelial Cells in Riverine Buffalo (Bubalus Bubalis)
Source: PLoS One. 2018 Jan 11;13(1):e0191380. doi: 10.1371/journal.pone.0191380 (PMC5764454; doi:10.1371/journal.pone.0191380)
Supplement: S2 Table — (DOCX) [file pone.0191380.s002.docx]

**Supplementary Table 2: EASE scores for affected GO terms in early and late time points post heat stress**

| **Category** | **Term** | **EASE**  **Score** | **Count** | **LT** | **PH** | **PT** | **p-value** | **Benjamini** |
| --- | --- | --- | --- | --- | --- | --- | --- | --- |
| **Early-up** | | | | | | | | |
| Biological_process | regulation of cell cycle | 1.08 | 6 | 67 | 261 | 8785 | 4.6E-2 | 1.0E0 |
|  | electron transport chain | 0.99 | 3 | 67 | 87 | 8785 | 1.4E-1 | 1.0E0 |
|  | regulation of apoptosis | 0.83 | 6 | 67 | 295 | 8785 | 7.0E-2 | 1.0E0 |
| Molecular_function | transcription factor binding | 0.72 | 5 | 64 | 127 | 9249 | 2.1E-1 | 9.9E-1 |
|  | chaperon activity | 0.72 | 7 | 64 | 715 | 9249 | 3.6E-1 | 1.0E0 |
| Cellular_component | mitochondrion | 0.99 | 12 | 55 | 731 | 6966 | 2.2E-2 | 9.6E-1 |
|  | plasma membrane part | 0.5 | 5 | 55 | 715 | 6966 | 8.2E-1 | 1.0E0 |
| **Early down** | | | | | | | | |
| Biological_process | cell surface receptor linked signal transduction | 1.76 | 27 | 217 | 1450 | 8785 | 9.7E-1 | 1.0E0 |
|  | oxidation reduction | 1.73 | 10 | 192 | 370 | 7349 | 6.3E-1 | 9.8E-1 |
|  | response to stimulus | 1.55 | 9 | 217 | 136 | 8785 | 1.9E-2 | 9.8E-1 |
|  | sensory perception | 1.55 | 7 | 217 | 134 | 8785 | 1.1E-1 | 9.7E-1 |
| Molecular_function | binding | 1.73 | 11 | 221 | 233 | 9249 | 5.2E-2 | 7.9E-1 |
|  | receptor activity | 1.07 | 5 | 221 | 51 | 9249 | 3.3E-2 | 7.5E-1 |
| Cellular_component | extracellular region | 2.2 | 39 | 173 | 892 | 6966 | 4.3E-4 | 9.5E-2 |
|  | Plasma membrane | 1.55 | 40 | 173 | 1186 | 6966 | 3.2E-2 | 5.0E-1 |
|  | cell-cell junction | 1.3 | 7 | 173 | 93 | 6966 | 2.7E-2 | 5.1E-1 |
| **Late up** | | | | | | | | |
| Biological_process | negative regulation of apoptosis | 0.89 | 4 | 66 | 146 | 8785 | 9.3E-2 | 1.0E0 |
|  | cell cycle | 0.75 | 5 | 70 | 149 | 7349 | 1.6E-1 | 1.0E0 |
| Cellular_component | cell fraction | 0.63 | 4 | 47 | 237 | 6966 | 2.1E-1 | 1.0E0 |
| **Late down** | | | | | | | | |
| Biological_process | Immune system response | 0.9 | 15 | 54 | 261 | 8785 | 5.9E-2 | 3.1E-1 |
| Cellular_component | cytoskeleton | 0.9 | 10 | 40 | 575 | 6966 | 8.3E-2 | 3.1E-1 |
